# Predicting the three-dimensional folding of *cis*-regulatory regions in mammalian genomes using bioinformatic data and polymer models

**DOI:** 10.1186/s13059-016-0909-0

**Published:** 2016-03-31

**Authors:** Chris A. Brackley, Jill M. Brown, Dominic Waithe, Christian Babbs, James Davies, Jim R. Hughes, Veronica J. Buckle, Davide Marenduzzo

**Affiliations:** SUPA, School of Physics and Astronomy, University of Edinburgh, Mayfield Road, Edinburgh, EH9 3JZ UK; MRC Molecular Haematology Unit, Weatherall Institute of Molecular Medicine, Oxford University, Oxford, OX3 9DS UK; Wolfson Imaging Centre Oxford, Weatherall Institute of Molecular Medicine, Oxford University, Oxford, OX3 9DS UK

**Keywords:** Chromosome conformation, Polymer model, Fluorescence *in situ* hybridization, cis-regulation

## Abstract

The three-dimensional (3D) organization of chromosomes can be probed using methods like Capture-C. However, it is unclear how such population-level data relate to the organization within a single cell, and the mechanisms leading to the observed interactions are still largely obscure. We present a polymer modeling scheme based on the assumption that chromosome architecture is maintained by protein bridges, which form chromatin loops. To test the model, we perform FISH experiments and compare with Capture-C data. Starting merely from the locations of protein binding sites, our model accurately predicts the experimentally observed chromatin interactions, revealing a population of 3D conformations.

**Electronic supplementary material:**

The online version of this article (doi:10.1186/s13059-016-0909-0) contains supplementary material, which is available to authorized users.

## Background

The three-dimensional (3D) spatial organization of mammalian chromosomes in vivo is a topic of fundamental importance in cell biology [1–5]. Understanding how chromatin conformation becomes modified on a local scale to up-regulate transcription from genes during differentiation or development is critical not only to decipher a fundamental biological process, but also to delineate the role this process may play in human disease and potential therapies. The higher scale organization of chromatin in the nucleus also has important roles to play in this regard [5–9], as the spatial structure of chromosomes is tightly linked to transcription. For instance, active genes can cluster at nuclear speckles [10, 11]; conversely peripheral lamina-associated domains comprise regions of the DNA that are not generically transcriptionally active [12, 13]. The 3D structure of the genome is, therefore, intimately related to its function.

Thanks to the development of high-throughput experimental techniques based on chromosome conformation capture (3C) [1], such as Hi-C and Capture-C [2–4, 14, 15], it is now possible to probe experimentally which regions of the genome of a given cell type are spatially proximate in vivo. A major result obtained with these methods has been the discovery that chromosomes are organized in a series of topologically associated domains (TADs) [2–4], which are separated by boundaries, but whose biological nature remains elusive. While the TAD boundaries are thought to be largely conserved across cell types, the arrangement of the chromatin within a TAD is not [16]. This internal organization depends on the activity of the genes within a domain, and is likely related to the action of *cis*-regulatory elements [DNA regions where the binding of a transcription factor (TF) can regulate the expression of a gene that is tens or hundreds of kilobase pairs (kbp) away] [17, 18].

The pattern of interactions revealed by most 3C-based experiments is an average over a large population of cells, yet it has become clear that there is a remarkable variability in both chromosomal conformation and chromatin interactions between different cells [19, 20]. Thus, it is an important challenge to understand how the chromosome conformation in single cells leads to the observed population average, and to decipher the mechanism underlying such arrangements. To address this issue, here we present an in silico investigation of the local folding and resulting interaction maps of important active gene loci in mouse erythroblasts. We concentrate on the well-studied *α* and *β* globin loci, which have long been model systems for understanding *cis*-regulatory interactions [14, 21–30]. These loci are known to have tissue-specific organization, and expression of the different genes within the loci varies through development and erythropoiesis. As a comparison, we also study embryonic stem cells where these genes are not active. Our main result is that our model predicts patterns of contacts that are close to that found by high-resolution Capture-C experiments, reproduces the changes in such patterns following differentiation, and explains existing observations on the biology of the globin loci in mouse. Our predictions also compare favorably with new fluorescence in situ hybridization (FISH) experiments that give spatial separation measurements between specific genomic locations in individual cells. This level of agreement is especially remarkable because it essentially involves no fitting.

Our model builds on the minimal assumption that the spatial organization of eukaryotic chromosomes is maintained largely through the action of proteins or protein complexes, which can form bridges by simultaneously binding to more than one site in the genome, and forming loops from the intervening chromatin [4, 31–36]. We treat the chromatin fiber as a simple bead-and-spring polymer (Additional file 1: Figure S1), and coarse-grain the bridge-forming protein complexes into single units. We then “paint” the polymer according to bioinformatic data characterizing protein binding and chromatin state in the relevant cell type, and use molecular dynamics to simulate the motion of the region of interest (see Additional file 1: Figure S1 for a schematic diagram and Additional file 2: Supplementary Methods for the full details of the model). The chromatin fiber and proteins diffuse as though subject to the thermal fluctuations of the nucleoplasm; the protein complexes can bind and dissociate from the chromatin and form bridges, and the fiber adopts conformations that are consistent with the entropic and energetic constraints of the system. By repeatedly running the simulation with different random thermal motions, we can generate a population of equilibrium conformations representing a population of cells. Some examples of other studies where polymer models have been applied to study chromatin are [20, 31–34, 37–40].

To keep our model as simple as possible, we use the locations of DNase1 hypersensitive sites (DHSs) as a proxy for binding sites of a generic type of protein bridge, which we imagine is made up from complexes of TFs and other DNA-binding proteins. The choice of DHSs as binding sites is justified due to their well-documented tendency to correlate with open chromatin, or euchromatin, and with peaks in ChIP-seq data for many TFs [41], such as GATA1, Nfe2 Scl/Tal1 and Klf1, all of which are known to be important for globin regulation (see Additional file 3: Figure S2). The interactions between the many TFs and co-factors that might form the bridging complexes involved in *cis*-regulatory binding are not well characterized, and the DHS approximation avoids the need to make any assumptions. One factor that most certainly has a chromatin architectural role is the CCCTC-binding factor (CTCF) [4, 35, 40, 42–44]. This protein is thought to form dimers that drive looping between some of its specific binding sites scattered along the chromosomes of eukaryotic organisms. In particular, convergent CTCF binding sites have been proposed to delimit the extent of chromatin domains, which might be extruded through a looping complex, possibly comprising cohesin [40, 44, 45]. CTCF is, therefore, a bridge with an architectural role, and has, indeed, been dubbed a global genome organizer [4, 35, 42]. Interestingly, chromatin has been found to compact on depletion of RAD21 and CTCF [37]. To reflect its perceived importance, we treat CTCF proteins as separate bridges in the simulations; in this case, the binding sites are placed at peaks in the ChIP-seq data for CTCF binding (see Additional file 3: Figure S2). Our model, therefore, includes two species of putative protein bridges, which we denote CTCF and DHS binding proteins (or bridges), respectively. Furthermore, we consider the hypothesis that some histone modifications (e.g., H3K4 monomethylation at enhancers or trimethylation at active promoters) act to recruit bridging proteins [46]. We include this in the model by introducing a weaker, non-specific interaction between the bridges and H3K4me1 modified regions (which are not already labeled as CTCF or DHS bridges); since the hypersensitive sites at regulatory elements are often surrounded by H3K4me1 modified regions, these act as a funnel, which effectively directs proteins to their high affinity binding sites [47].

## Results

### Chromatin folding in the mouse ***α*** globin locus

**Fig. 1:**
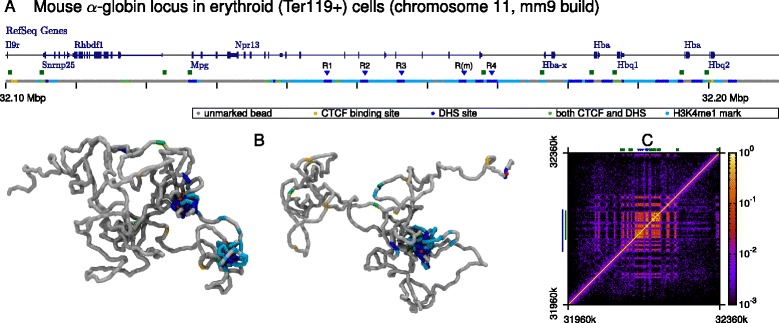
Simulating the *α* globin locus. **a** Browser view showing genes in the vicinity of the *α* globin locus, alongside a schematic indicating the coarse-graining used in the simulations. A 110-kbp section of the 400-kbp chromatin fragment that was simulated is shown. As described in the text, simulation chromatin beads were designated as CTCF binding sites, DHS binding, H3K4me1 modified sites and combinations of these. The positions of the set of five regulatory elements are indicated with *blue triangles* and promoters with *green squares*. **b** Example simulated configurations of the locus. CTCF proteins (*green*) and DHS binding proteins (*red*) are shown; the chromosome fragment is colored as in (**a**). See also Additional file 4: Video S1 for a 3D view of the configurations, from which the CTCF proteins are more readily visible. Parameters for the polymer model and the bridge–chromatin affinity are given in full in Additional file 2: Supplementary Methods. **c** Contact map showing the frequency of contacts between each chromatin bead in 1000 simulated configurations. Note that the *color bar* shows a logarithmic scale. The *blue line* to the *left* indicates the region that is shown in (**a**). The *green line* to the *left* indicates the region that is used for the clustering analysis (Fig. 2 and text)

First, we use our model to predict the folding of a 400-kbp region around the mouse *α* globin locus (chr11:31960000–32360000, mm9 build; each polymer bead represents 400 bp, or two nucleosomes, see Fig. 1a and Methods). This well-studied cluster contains five globin-related genes: the *ζ* globin gene (*Hba-x*, expressed in embryonic erythroid cells, but silent in adult cells), two copies of the *α* globin gene (*Hba*, expressed in fetal and adult erythroblasts) and two *θ* globin genes (*Hbq1* and *Hbq2*, only weakly expressed in adult tissue). Expression of the genes in the cluster is controlled by several regulatory elements: the multi-species conserved elements R1–4 and the mouse specific R(m). Some of these are contained within the introns of *Nprl3*, one of several widely expressed genes that surround the locus; the R2 element (known as HS-26 in mouse and equivalent to HS-40 in human) is thought to be particularly important for globin regulation [21, 23, 27]. Figure 1a shows the binding sites for CTCF and DHS across the region considered (informed by ChIP-seq and DNase-seq data for adult erythroid cells – see Additional file 3: Figure S2); the positions of the H3K4me1 methylation marks are also indicated (from ChIP-seq data for the same cell type, see Additional file 3: Figure S2). In our simulations, proteins bind strongly to the CTCF or DHS labeled beads, and also weakly to the H3K4me1 marks. Some typical snapshots from our simulations are shown in Fig. 1b and Additional file 4: Video S1 (CTCF and DHS binding proteins are shown as red and green spheres, respectively), while the average contact map is shown in Fig. 1c.

**Fig. 2:**
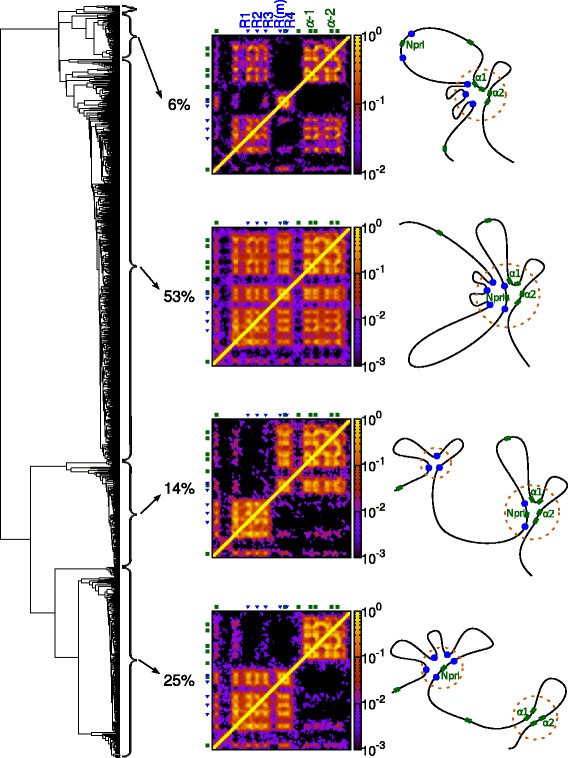
Conformations of the *α* globin locus can be grouped by similarity. A clustering analysis gives a dendrogram (*left*), which indicates how similar or different the conformations are. Conformations fall into four main representative structures depending on the pattern of contacts they exhibit (see Additional file 2: Supplementary Methods). Contact maps for each representative structure are shown (*center*; the region shown is indicated by the *green line* in Fig. 1 c), as is a schematic of each representative structure (*right*). The proportion of simulated conformations adopting a given structure gives a prediction of the frequency with which that structure will occur in a population of cells

As anticipated, one of the main strengths of our approach is that it naturally outputs information on each member of the population of chromatin conformations (these can be thought of as representing different cells, or the same cell at different times), which we can then further interrogate. A clustering analysis (i.e., grouping the conformations by similarity; see Additional file 2: Supplementary Methods for details) of 1000 simulated conformations reveals that the locus folds into four main representative structures (Fig. 2). The main distinction between these structures is whether a single bridging-induced globular domain forms (of size ∼70 kbp), or whether it breaks into two smaller microdomains, one containing around 40 kbp, and the other one around 25 kbp. The size of these globular microdomains does not exceed 100 kbp, so these are much smaller than TADs (the median size of a TAD is 1 Mbp [3]); interestingly, though, their size is comparable to that of the sub-TAD domains observed within active regions [4], and also to that of the so-called supercoiling domains recently found in mammalian cells [48].

In the most common representative structure, which accounts for 53 % of the total observed conformations for the locus, there is a single globular domain containing the promoters of the globin genes, the promoters of the two neighboring genes *Mpg* and *Nprl3*, and all five known regulatory elements. A similar representative structure, which accounts for 6 % of conformations, also has a single globular domain, but the region that contains the *Nprl3* promoter is in a loop outside the globule. A third representative structure accounts for 14 % of the conformations: here two globular microdomains form, where the *α* genes interact with only the two genomically closest regulatory elements. The fourth structure, which is adopted by about 25 % of the conformations, has again two microdomains, but their composition is different: now the *α* genes are no longer in the same microdomain as the regulatory elements. We expect that these genes should be transcriptionally inactive when the locus adopts this structure. Finally, there are a small number (∼1 %) of conformations that do not fit into any of these four clusters. It is also interesting to note that the *ζ* gene and *Mpg* seldom interact with the elements (these genes are not widely expressed in adult erythroid cells). The arrangement within the domains can be further probed by looking at which promoters are directly interacting with the different regulatory elements in each conformation (see Additional file 5: Figure S3). We find, for example, that one or more of the *α* promoters interacts with one or more of the elements in 65 % of conformations, and that *Hba-a1* interacts with the elements in 53 % of conformations whereas *Hba-a2* interacts in only 41 %. This is qualitatively consistent with experiments in which mRNA expression from the two *α* globin paralogues was measured independently (on the basis of 3 ^′^ sequence divergence), which showed that the gene situated linearly closer to the enhancer elements, *Hba-a1*, is always expressed at a higher level [26].

**Fig. 3:**
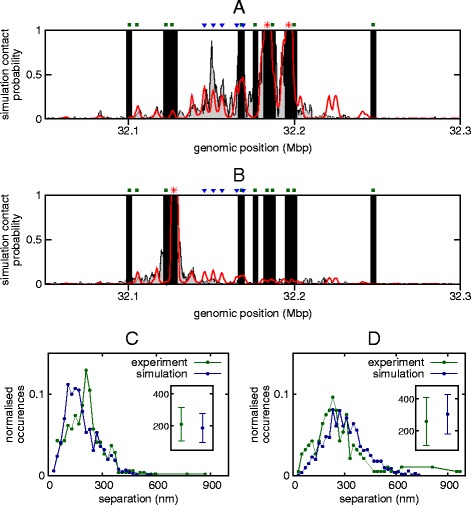
Simulations compare favorably with experimental data. **a** Plot showing the contacts made with the promoters of the two *α* globin genes (locations indicated by *red asterisks*; the positions of the regulatory elements and other gene promoters are also indicated). Simulation results (*red*) are shown alongside Capture-C data (*gray*); in both cases the plots show the contacts to both genes combined (since each copy of the gene has the same sequence it is impossible to separate these in the experiment). *Black bars* indicate regions where there is no contact data (i.e., between captured regions; see Additional file 2: Supplementary Methods and Ref. [14]). Since Capture-C data only give relative contact strength, the height of the experimental data has been scaled so as to best fit the simulation results (see Additional file 2: Supplementary Methods). **b** As in (**a**), but now showing the contacts made with the *Mpg* promoter (position indicated by *red asterisk*). Although *Mpg* is roughly the same genomic distance away from the regulatory elements as the *α* globin genes, it interacts with them less frequently. **c** Plot showing the distribution of the 3D separation of the *α* globin promoters and the probe pE located at the regulatory elements R1–3. Simulations are compared with FISH measurements (see Methods and Additional file 7: Figure S5) performed on mature erythroblasts 30 hours after differentiation, when the globin genes are maximally expressed. The inset shows the mean and standard deviation for each case. **d** As in (**c**), but showing the separation of the *α* promoters and a downstream control probe p58 located within the *Sh3pxd2b* gene

Importantly, we can also compare the interactions predicted by our simulations with recent high-resolution Capture-C data [14], which mapped the chromosomal contacts within a number of *cis*-regulatory landscapes in mouse erythroblasts (see Additional file 2: Supplementary Methods). Specifically, Fig. 3a compares Capture-C and in silico patterns of contacts with the promoters of the two *α* globin paralogues (which cannot be separated in the experimental data as they share the same sequence). Figure 3b shows a similar plot for the *Mpg* promoter. The results show that, remarkably, *with the sole input* of the ChIP-seq and DNase-seq data giving the locations of the protein binding sites, we can reproduce to a good accuracy the Capture-C profiles. In particular, we reproduce the contacts between the *α* promoters and the five known regulatory elements; we also reproduce that there is some interaction between the regulatory elements and the *Nprl3* promoter (see Additional file 6: Figure S4), but far fewer interactions with the *Mpg* promoter, despite the fact that this gene is a similar genomic distance away from the elements as the *α* genes.

To assess further the level to which the population of locus conformations predicted by our model gives a faithful representation of the organization of the *α* globin locus in real cells, we performed FISH experiments (see Methods) to obtain distributions of the separations of probes at different positions across the locus. These measurements also allow us to parametrize the physical size of the 400-bp simulation beads by fitting the means of each distribution (see Methods and Additional file 7: Figure S5); this is the only fitted parameter in our model, and the fit yields a size of 15.8 nm, which is reasonable given that 400 bp corresponds to two nucleosomes. Plotting the experimental and simulation separation distributions on the same axes (Fig. 3c–d, and Additional file 7: Figure S5d–g) reveals that once more the simulations give an accurate prediction of the structure of the locus; for example, the separation of the *α* promoters and pE at the regulatory elements R1–3 shows a narrow distribution peaked about a mean value of ∼200 nm, whereas the separation of the promoters and a probe p58 at roughly the same genomic distance, but telomeric to the locus, shows a much broader distribution with a mean closer to 300 nm.

We can also define a quantitative score $\mathcal {Q}$, taking values between 0 and 1, which indicates how well our simulations predict the experimental Capture-C interaction profiles (see Additional file 2: Supplementary Methods for details). By combining Capture-C data from a number of promoters across the locus, we can obtain a mean $\mathcal {Q}$ value along with a standard error (Additional file 8: Figure S6). This allows us to compare results from different model set-ups. Specifically, we examined the effect on the experiment-simulation comparison scores of changes in: (i) chromatin stiffness, (ii) number of bridges and (iii) level of coarse-graining (see Additional file 2: Supplementary Methods and Additional file 8: Figure S6). For the first two cases, we find only a modest effect on the $\mathcal {Q}$-score for the simulated configurations (Additional file 8: Figure S6); if we decrease the resolution of our model by changing the coarse-graining, then this performs less well. Interestingly, the representative structures found from the clustering analysis of the population of conformations found in silico are always the same. What changes in some cases is the proportion of conformations that adopt each representative structure. In the model where the chromatin fiber was stiffer, the globular microdomain structure containing all of the regulatory elements occurred less often, whereas the structure where the *Nprl3* promoter loops out was more likely; this is because holding the *Nprl3* promoter in the microdomain requires bending of the chromatin fiber, which is disfavored when this is stiff. Also, when we examined the effect of changing the number of protein complexes in the simulations, we found that, as more proteins are introduced, there is a greater likelihood that the locus adopts a structure with two globular microdomains; this is because forming more protein bridges between chromatin binding regions, while being energetically favorable, leads to the formation of more loops whose entropic cost increases non-linearly with the number of loops [49].

### Chromatin folding of the ***β*** globin locus

**Fig. 4:**
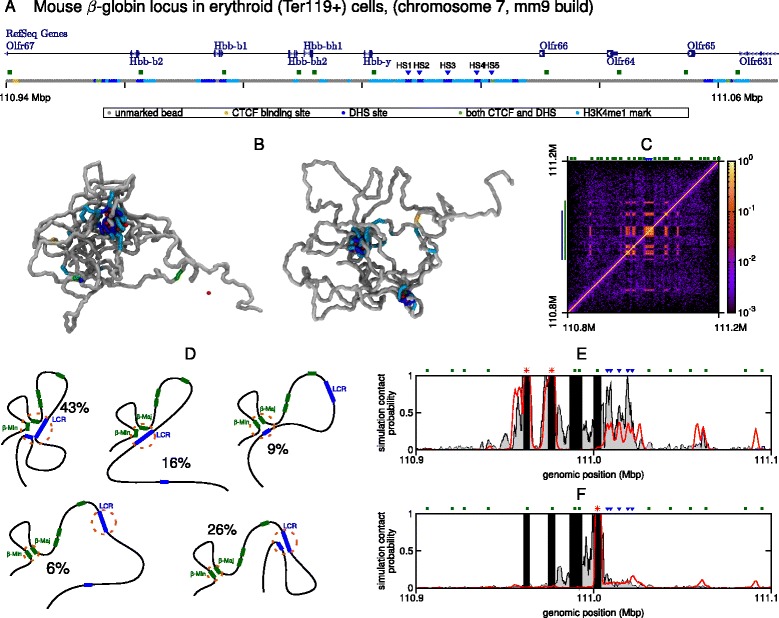
*Cis*-interactions of the *β* globin locus. **a** Browser view showing genes in the vicinity of the *β* globin locus, alongside a schematic indicating the coarse-graining used in the simulations. A 130-kbp section of the 400-kbp chromatin fragment that was simulated is shown. The positions of the known regulatory elements within the LCR are indicated with *blue triangles* and promoters with *green squares*. **b** Example simulated configurations of the locus. CTCF proteins (*green*) and DHS binding proteins (*red*) are shown; the chromosome fragment is colored as in (**a**). **c** Contact map showing the frequency of contacts between each chromatin bead in 500 simulated configurations. The *color bar* shows a logarithmic scale. The *blue line* to the *left* indicates the region that is shown in (**a**); the *green line* indicates the region that is used in the clustering analysis. **d** As in Fig. 2, clustering analysis allows conformations to be grouped by their structural features. Schematics of the representative structures are shown, with the percentage of conformations in which they occur. A dendrogram and contact maps for each representative structure are shown in Additional file 10: Figure S8. **e** Plot showing the contacts made with the promoters of the two *β* genes (locations indicated by *red asterisks*; the positions of the regulatory elements and gene promoters are indicated). Simulation results (*red*) are shown alongside Capture-C data (*gray*); both cases show the contacts to both genes combined (since each copy of the gene has the same sequence it is impossible to separate these in the experiment). *Black bars* indicate regions where there is no contact data (see Ref. [14] and Additional file 2: Supplementary Methods). **f** Similar plot showing the contacts made with the *Hbb-y* gene (position indicated by *red asterisk*)

We also applied our chromosome-and-bridges model to the mouse *β* globin locus (chr7:110800000–111200000, mm9 build; Fig. 4, Additional file 9: Figure S7, and Additional file 10: Figure S8). This locus contains five globin genes: the *ε*y gene, *β*h1 and 2, and two *β* globin genes *β*-Major and *β*-Minor. The expression of each gene depends on the stage of development (the *ε*y and *β*h1 genes are predominantly expressed in embryos, while the *β* genes take over in adults), and is controlled by interactions with a series of DHSs in a region known as the locus control region (LCR) [21, 24]. Unlike the *α* globin locus, the *β* globin genes are surrounded on either side by a condensed chromatin region, containing genes that are not expressed in erythroid cells. As with the *α* globin case, we use ChIP-seq and DNase-seq data to label a bead-and-spring polymer that represents the gene locus (see Fig. 4a, and Additional file 9: Figure S7). A clustering analysis of a population of 500 simulated conformations reveals that the most abundant representative structure of the *β* globin locus (43 % of the total conformations, see schematics in Fig. 4c and dendrogram in Additional file 10: Figure S8) features a single globular domain, where the *β* Major and Minor promoters co-localized with the five regulatory elements in the LCR, and with a CTCF site on the telomeric side near the *Olfr65* gene. A further 16 % of conformations adopt a similar representative structure, but the promoters interact only with the LCR. We also note that when the locus adopts these structures, there is an interaction between the CTCF sites in the LCR and the one on the centromeric side of the *β* genes near the *Olfr67* gene (these contacts are just visible on the left and bottom edges of the top two contact maps in Additional file 10: Figure S8a), which has previously been observed in both definitive erythroblasts and erythroid progenitors, but is absent in non-erythroid tissue [22, 24]. This is consistent with the hypothesis that CTCF-mediated loops in progenitors hold the locus in a structure poised to facilitate *β* globin expression upon differentiation [24] (though see below). A third representative structure, which accounts for 9 % of the simulated conformations, has the *β* promoters interacting only with the DHS near *Olfr65*. The Capture-C data, along with previous work [22, 24], confirm the prediction that this site (usually denoted HS-60) interacts with the *β* globin promoters; indeed, it has been previously shown that there are interactions between all hypersensitive sites in the locus [22] and the pair of sites HS-60/-62 are normally taken to demarcate the boundary of the locus. Whether this particular DHS (HS-60) has enhancer properties remains unclear; however, it binds Scl/Tal1 (a TF thought to play a key role in hematopoietic differentiation [50]), is near to a CTCF binding site (HS-62), and is within a region marked by monomethylation of histone H3 Lys4, which is normally associated with enhancers. In the remaining 32 % of the conformations (bottom two schematics in Fig. 4d), the *β* globin promoters are still together, but do not interact with the hypersensitive sites (Additional file 10: Figure S8a).

We note that the microdomains that form in each type of the five representative structures have more looped out regions (consistent with conclusions from 3C experiments in Ref. [22]) than in the *α* globin locus (compare contact maps in Figs. 1c and 2 with Fig. 4c and Additional file 10: Figure S8a – more gaps are seen between the blocks of highly probable interactions in the *β* globin case). This indication that the *β* globin locus is less compact than the *α* globin case is borne out in measurements of the overall 3D size of the simulated loci (see distributions of the radius of gyration of the polymer in Additional file 11: Figure S9g compared to the *α* globin case in Fig. 7g).

As for the *α* globin locus, our simulations predict contact patterns that are in good agreement with Capture-C data, both for the *β* Major and Minor gene promoters (Fig. 4e) and for the *Hbb-y* promoter (Fig. 4f). This demonstrates that our model is not gene-specific, but can be applied, in principle, genome-wide, at least to active regions; the two bridges that we model, CTCF and DHS binding proteins, are, indeed, found in most euchromatic, open chromatin, regions. Given its relatively low computational cost (harvesting 500 conformations for a 400-kbp chromosome region at a 400-bp resolution can be done in about a day with a multi-core machine, see Additional file 2: Supplementary Methods), we expect this modeling to be useful in predicting the overall folding of previously uncharacterized active chromosomal loci – the knowledge of the predicted population of 3D structures can then direct further high-resolution Hi-C, Capture-C or fluorescence hybridization experiments (as in Figs. 3 and 4e, f) to characterize that region more accurately.

### The model accurately reproduces differences in locus folding across cell types

**Fig. 5:**
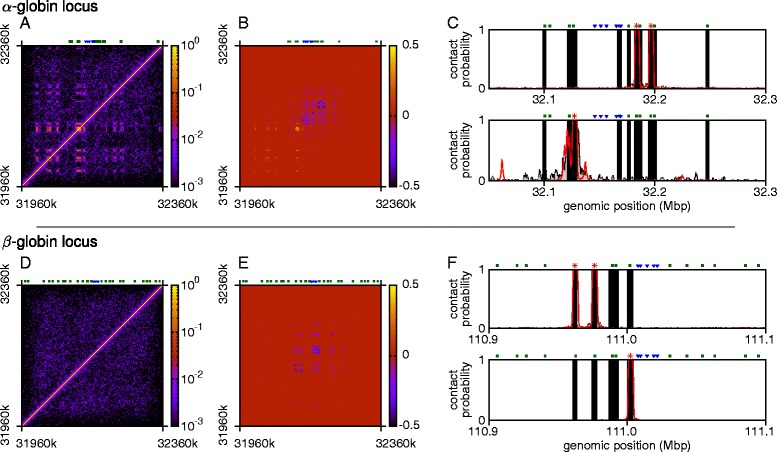
Simulations show changes in locus organization across cell types. **a** Contact map for 500 conformations for the *α* globin locus in mES cells. Simulations are performed as in Fig. 1, but using mES cell ChIP-seq and DNase-seq data, as shown in Additional file 11: Figure S9. **b** Difference between the contact maps in panel (**a**) and Fig. 1 c. *Blue regions* indicate contacts that were present in erythroblasts, but not mES cells, and *yellow* indicates contacts present in mES cells but not erythroblasts. **c** Plots comparing simulations and Capture-C data for MESs (data from Ref. [14]). **d**–**f** Similar plots but for the *β* globin locus

Importantly, because data showing protein binding, hypersensitive sites and histone modifications are available for different cell types, we can also predict changes in the 3D organization of a chromosomal region across cell types or at different times in development. We show in Fig. 5 how the folding of the globin loci differs in mouse embryonic stem (mES) cells (where the globin genes are inactive) with respect to the organization predicted for erythroblasts. The bioinformatic data used to inform our modeling for stem cells are given in Additional file 12: Figure S10.

Figure 5a shows the contact map predicted from simulations of the *α* globin locus. Our model predicts that in embryonic stem cells the contacts are much sparser than in erythroblasts, that the bridging-induced domain around the *α* globin gene is lost (Fig. 5b), and that no interactions with the regulatory elements are observed; the same is true of the neighboring *Mpg* promoter. Once again, the contacts observed in silico reproduce the experimental ones (Fig. 5c), with some minor inaccuracies for *Mpg* (which likely originate from our approximation that all DHSs are the same in regards to bridge formation, but nevertheless highlight the principle that the locus can adopt a completely different shape in a different cell type). When repeating the analysis for the *β* globin locus, we find that the loss of non-local contacts is even more dramatic (Fig. 5d, e), and the agreement with the data even more remarkable (Fig. 5f), with all non-local (i.e., off-diagonal) interactions being absent.

**Fig. 6:**
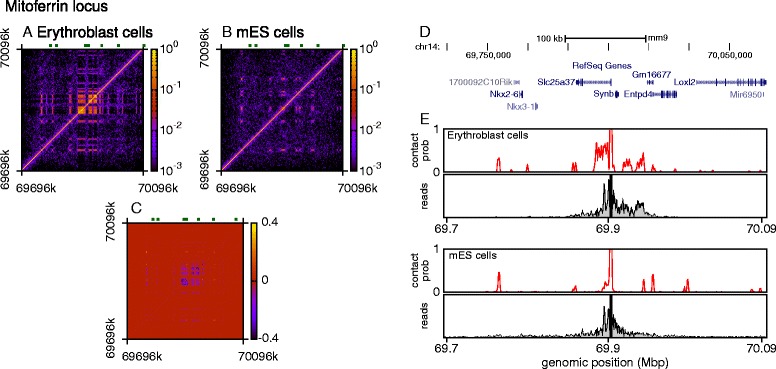
Simulations also correctly predict looping for a less studied locus. Simulations of the *Slc25a37* gene (Mitoferrin1) were performed for mouse erythroblasts and embryonic stem cells, using similar input data as for the globin loci (DNase-seq, and ChIP-seq for CTCF and the H3K4me1 histone modification). **a** Contact map from the simulations of erythroblasts showing the frequency of contacts between each chromatin bead in 500 simulated configurations. **b** Similar contact map for the same locus in mES cells. **c** Difference between the contact maps in panels (**a**) and (**b**). *Blue regions* indicate contacts that were present in erythroblasts, but not mES cells, and *yellow* indicates contacts present in mES cells but not erythroblasts. **d** Browser view showing the genes across the 400-kb simulated region. **e** Plots showing the interaction profiles for the *Slc25a37* promoter in each cell type, comparing simulation results (*upper panels*) with new Capture-C data (*lower panels*). Note that the genomic coordinates are aligned with the browser view in (**d**)

To demonstrate further the wide applicability of the model, we also perform a set of simulations for a region surrounding the *Slc25a37* (Mitoferrin1) gene in both mouse erythroblasts and embryonic stem cells. This gene encodes a mitochondrial protein essential for iron import into mitochondria; however, much less is known about this locus than about the *α* or *β* globin, and so our results represent a true prediction of its folding. The input data used were similar to that of the globin loci, and are given in Additional file 13: Figure S11. As shown in Fig. 6, the simulations predict that in the erythroid cells (where the gene is active) the locus forms a compact domain around *Slc25a37* and *Entpd4*; the *Slc25a37* promoter interacts strongly across the *Slc25a37* gene, but also with two distinct regions between the nearby *Synb* and *Gm16677* genes (Fig. 6e top panel). These are enriched for monomethylation of lysine 4 of histone H3 (see Additional file 13: Figure S11d), suggesting that sites within these regions have enhancer activity (as was also proposed in Ref. [51]). To test these predictions, we compare with new Capture-C experiments (performed as detailed in Ref. [14]). As before, our very simple model gives a remarkable agreement with the data: strong interaction with the putative enhancer regions is observed in the erythroid, but not the stem cells. Some longer distance interactions that are predicted in both cell types are not found in the experimental data; these errors are due to our approximation that bridges can form between any DNase hypersensitive sites, and the agreement would likely be improved with a different choice of input data (e.g., using TFs involved in regulation of this gene).

### The typical 3D structures of the globin loci are preserved in CTCF or other TF knock-outs

Another strength of our approach is that it is easy to alter the protein binding profiles in our simulations to investigate, e.g., genome modifications or protein knock-outs etc., and predict the consequences of these for the 3D organization in vivo. For example, we can switch off interactions with the hypersensitive sites, and only include the CTCF bridges in the simulation, or simulate a CTCF knock-out by switching off interactions with the CTCF sites and any hypersensitive sites where only CTCF binds (i.e., DHSs that bind CTCF, but none of the other TFs implicated in globin regulation).

**Fig. 7:**
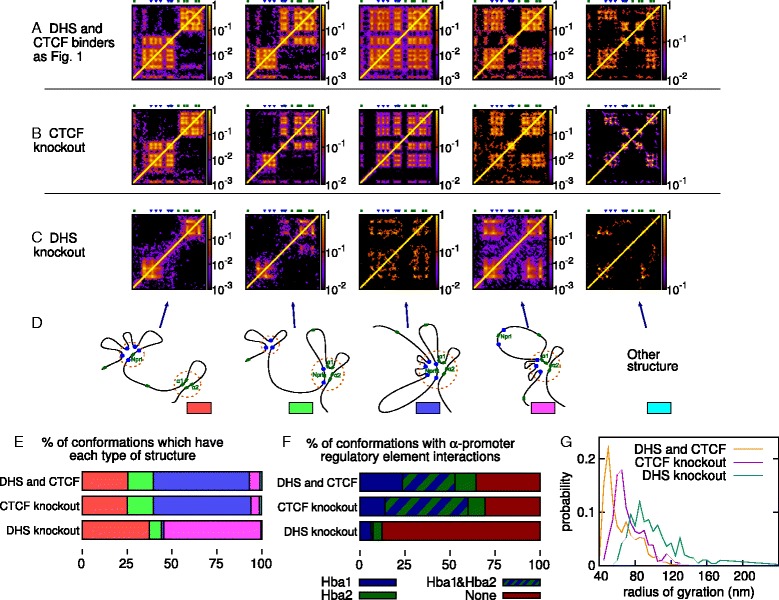
Simulations predict the effect of protein knock-outs in the *α* globin locus. Plots showing the effect of a CTCF knock-out and a DHS knock-out (equivalent to knocking out all protein complexes involved in looping the *α* globin locus *except* CTCF). **a**–**c** Contact maps showing the interactions between different chromosomal locations for conformations within each group identified by clustering analysis. Maps from three sets of simulations are shown; the positions of the known regulatory elements and gene promoters are indicated above each plot. **d** Schematics showing the structure of the locus within each group. **e** Plot showing the percentage of conformations that belong to each group identified by the clustering analysis. The color key is given in (**d**). **f** Plot showing in what percentage of conformations the two *α* globin gene promoters are interacting with one or more of the known regulatory elements. **g** Plot showing the distribution of the radius of gyration of the locus across the simulated conformations. The radius of gyration is defined as ${R_{g}^{2}}=(1/N) \sum _{i=1}^{N} (\mathbf {r}_{i}-\bar {\mathbf {r}})^{2}$, where **r**_*i*_ is the position of the *i*th chromatin bead in the polymer, and $\bar {\mathbf {r}}$ is the mean position of all *N* chromatin beads

For the *α* globin locus, we find that, surprisingly, for both the CTCF and DHS knock-outs the same folded structures can still form (Fig. 7a–d). For the CTCF knock-out, the relative proportions of each structure found in the clustering analysis remain largely unchanged (Fig. 7e): the most common one is again the single globular domain containing the *α* promoters and all regulatory elements. If we assume that the level of *α* globin expression correlates with the fraction of conformations in which one or more of the *α* promoters is interacting with one or more of the regulatory elements, then this expression level also remains largely unchanged (the genes are active in 65–70 % of conformations; see Fig. 7f). For the DHS knock-out on the other hand, the number of conformations showing regulatory element interactions drops to less than 20 %. There is also a change in the proportions of the different groups found by the clustering analysis, with the structure in which the *Nprl3* promoter loops out of a single domain becoming most common. Nevertheless, it is remarkable that despite loss of binding at the regulatory elements (which presumably reduces *α* globin expression), the CTCF sites near the *Hbq1* and *Hbq2* promoters, and within the introns of the *Nprl3* gene (green and yellow in Fig. 1a) are sufficient to allow the locus to fold into the same representative structures. We can also measure the effect on the overall size of the domain by calculating the radius of gyration of the polymer; Fig. 7g shows the distribution for each of the in silico knock-outs. We see that loss of protein binding generally leads to an expansion of the locus, with the DHS knock-out having more effect than the CTCF case.

A similar scenario applies to CTCF and DHS knock-outs in the *β* globin locus (Additional file 11: Figure S9). Here, however, the contact map for each of the groups identified by the clustering analysis (Additional file 11: Figures S9a–c) shows some subtle differences between the knock-outs. Again the CTCF knock-out appears to have little effect, leading to only small changes in the fraction of simulations adopting each structure or the contacts between the *β* promoters and the LCR. The DHS knock-out leads to a notable reduction in the promoter–LCR interactions, and a reduction in the number of conformations adopting the structure where the *β* promoters interact with the hypersensitive site near the *Olfr65* gene. This locus also expands upon protein knock-outs, albeit to a lesser extent than the *α* globin case; this is probably due to the *β* globin locus being less compact initially.

Given the suggestion that CTCF proteins play a key role in genome organization, it might seem surprising that the knock-out simulation shows a relatively minor change in the folding structures and promoter–enhancer interaction in both globin loci. However, CTCF is known to have a variety of different functions; for instance, it acts as a barrier against the spreading of repressive heterochromatin, or as an insulator, preventing interactions with other nearby chromosome regions [42]. A recent study suggested that a depletion of CTCF has only a mild effect on the domain organization of chromosomes as found via Hi-C experiments [52], and a ChIA-PET analysis of the contacts made between CTCF-bound regions found that only a fraction of the 40,000 CTCF binding sites are involved in these [53]: presumably, this is related to the recently discovered importance of CTCF binding site directionality in loop formation [4, 43, 45]. In the specific case of the *β* globin locus, another recent study found that reducing the abundance of CTCF protein or disrupting a specific CTCF binding site within the locus in erythroid progenitor cells leads to a loss of chromosome looping; however, upon differentiation to mature erythroblasts, these cells are still able to express *β* globin, and fruitful interactions between the promoters and the LCR can still form [25] (i.e., setting up loops in progenitor cells appears not to be necessary). Together this suggests that the globin loci may be examples where CTCF-mediated chromosome loops are not crucial in determining the 3D organization, though, of course, CTCF is likely to have some other function (e.g., protecting other nearby genes from activation) and may still play an important organizational role at a larger scale [28]. In our simulations, the CTCF bridges certainly do form loops, but in their absence the overall folding patterns can be maintained by the other bridges.

## Discussion

In this work, we have shown that a minimal polymer model informed by large bioinformatic data sets on protein binding can successfully reproduce the pattern of Capture-C contacts observed in the well-studied *α* and *β* globin loci within mouse erythroblasts (a cell type where these genes are highly active), and also within the less understood *Slc25a37* (Mitoferrin1) locus. Our model is built on the hypothesis that there exist architectural protein bridges, which we assume are either CTCF or generic bridges made up by complexes of TFs and other DNA-binding proteins. The only inputs we require are ChIP-seq data for CTCF binding and the map of DHSs, which we take as a proxy for the location of the binding sites for the generic protein bridges (DHS bridges). Importantly, our approach differs from other recent polymer modeling studies that also have predictive power [20, 29, 36], in that it does not rely on fitting to pre-existing 5C or Hi-C data. Due to this feature, it can be applied to relatively poorly characterized loci (e.g., Mitoferrin1, see Fig. 6), for which only few data exist (e.g., DNase tracks); the model can then be developed when needed as more experimental data become available.

Our model generates a population of conformations, hence we can predict, for instance, the distribution of distances between selected targets on the globin locus. These results compare very favorably with our FISH measurements, which allow us to estimate the physical size of the beads in our coarse-grained polymer (or equivalently, the DNA packing density in the chromatin fiber in the globin locus; this is the only fitting parameter in our model). The packing we obtain (15.8 nm for 400 bp) is consistent with open chromatin, which is reasonable since the region we focus on is highly active.

That our model generates a population of conformations, rather than a single average conformation, is important because it gives an estimate of the stochasticity and fluctuations in in vivo 3D organization. A key result of our model is that the conformations of the loci we studied can be grouped into a handful of representative structures, which account for different fractions of the whole population. In both the *α* and *β* globin loci, the analysis suggests that there is a split in these structures between two main types: those in which there is a single globular domain that includes the active genes together with their regulatory elements, and those where the globule splits into two microdomains. The single globule structures are favored by bridging, while the competing structure requires less bending and looping, and costs less entropy. (This is because there are more ways to place two microdomains in space than there are for a single one, and also because the entropy of forming *n* loops in the same place scales non-linearly with *n* [49].) There is a subtle balance between these contributions, which are both of the order of a few *k*_*B*_*T*, therefore, both structures coexist in the population. A consequence of this is that the globin loci are naturally poised close to a transition between two different 3D folding phenotypes; because the competition between bridging and entropy is likely to be a generic feature, we suggest that the plasticity associated with this balance between competing effects may be an underlying principle in the organization of active regions genome-wide. This suggests that the cell could tip the balance one way or another by changing the abundance or specificity of bridges, or the properties of the fiber (e.g., by histone modification or chromatin remodeling).

In future work, it will be interesting to compare these predictions with experimentally determined chromatin dynamics through cell differentiation, for example, examining the *α* globin genes using techniques that permit imaging of the locus during erythroid differentiation in live cells. Another application of the work might be to provide some explanation of how the *Hba-x* gene is silenced in adult erythroblasts: in all of our predicted conformations, it does not contact the known enhancer elements nor the surrounding gene promoters. It may also be informative to repeat the modeling for primitive erythroblasts, when sufficient protein binding and DNase hypersensitive data become available for that cell type.

As we have seen, our model can be further exploited to predict the organizational consequence of the knock-out of proteins such as CTCF (or our generic DHS bridge). Similarly, one can perform an in silico experiment that follows the consequences of modifying some genomic region within a locus. An intriguing example is the deletion of the R2 (HS-26) hypersensitive site in the *α* globin locus, which has been shown experimentally to result in a 50 % reduction of *α* globin RNA levels [23] (a much milder phenotype than the severe *α* thalassemia that results from a deletion of the equivalent HS-40 element in humans [27]). Removing the R2 site in our simulation only leads to a ∼3 % reduction in the number of conformations where the *α* promoters interact with the remaining regulatory elements. We can make our model more complex by replacing DHS binding proteins with bridges that bind to specific TF binding sites. For instance, GATA1 and Klf1 are a minimal set of TFs (see Additional file 3: Figure S2) that can interact to form bridges between the *α* globin promoters and the regulatory elements, and that can discriminate between the different elements (i.e., GATA1 binds to R1–4 only, whereas Klf1 binds to R2, and the *α* promoters only). Thus, we use a model with three protein species, binding strongly to GATA1, Klf1 and CTCF sites, respectively (no longer considering hypersensitive sites), and weakly to H3K4me1 modified regions (using ChIP-seq data as shown in Additional file 3: Figure S2), and repeat the in silico R2 knock-out experiment (see Additional file 14: Figure S12). Quite remarkably, in a wild-type simulation, this more detailed model reproduces the differences in peak heights for interactions between the *α* promoters and elements R1–3 as shown in the Capture-C data (i.e., there is a higher probability of interaction with R2 than R1 or R3; Additional file 14: Figure S12a). For the R2 knock-out case, the three-bridge model shows a ∼20 % reduction in the number of conformations where the *α* promoters interact with the remaining regulatory elements (much closer to what might be expected given the experimentally observed effect on *α* globin RNA levels). Therefore, our approach can be generalized to accommodate more biological detail in a modular fashion, where this detail is known.

We anticipate that the main application of our in silico chromosome folding model will be to investigate regions of mammalian and other eukaryotic genomes that are currently poorly characterized. The approach relies only on DNase hypersensitivity and protein binding data, which are available genome-wide for many organisms and cell types. Our technique is fast and inexpensive, so that it can be used to predict the organization of a large number of wild-type and modified genomic loci prior to, for example, a combination of detailed Capture-C, 5C or FISH experiments, directing focus to those regions whose predicted structure was deemed to be of particular interest. The ease with which genome modifications can be incorporated makes it highly applicable for investigation of the effect on 3D chromatin structure of, for example, single nucleotide polymorphisms at enhancers, which have been implicated in many diseases.

In the present work, we focused on looping interactions within a gene locus, at a sub-TAD length scale. Polymer models, and the principle of protein bridges driving chromatin conformations, can easily be adapted to treat larger looping and organization at the chromosome and genome scale, and this will be the subject of a future study.

## Methods

### Polymer model and simulation scheme

The chromatin fiber is modeled as a simple coarse-grained bead-and-spring polymer, where each bead represents 400 bp of DNA, or roughly two nucleosomes. The positions of the beads are updated via a molecular dynamics scheme (Langevin dynamics) using the LAMMPS (Large-scale Atomic/Molecular Massively Parallel Simulator) [54] software. Pairs of beads adjacent along the polymer backbone interact via finitely extensible non-linear elastic springs, and the polymer is afforded a bending stiffness via a cosine interaction between triplets of adjacent beads. We choose parameters such that the persistence length is four beads, which is reasonable for euchromatin [55]. The beads also interact with each other via a Weeks–Chandler–Anderson potential, meaning they cannot overlap. Protein complexes are modeled as single spheres that interact with each other also via a Weeks–Chandler–Anderson potential (i.e., they have a steric interaction only). Each chromatin bead represents a region of the chromosome locus of interest, and is labeled as binding or not for the various protein species according to the input data. Proteins interact with chromatin beads labeled as binding via a shifted, truncated Lennard–Jones interaction that has short-range repulsive and longer-range attractive parts; they interact with non-binding chromatin beads again via the Weeks–Chandler–Anderson potential. Full details of all interaction potentials are given in Additional file 2: Supplementary Methods, and parameter values in Additional file 15: Table S1. As input to the model, we use ChIP-seq and DNase-seq data (see Additional file 3: Figure S2, Additional file 9: Figure S7 and Additional file 12: Figure S10; data from Refs. [14, 50, 56–58] as indicated in figure captions) to identify protein binding sites in the chromosome region of interest. Full details of the bioinformatics data analysis are given in Additional file 2: Supplementary Methods.

### Capture-C data

The Capture-C data shown in Figs. 3, 4 and 5 and Additional file 6: Figure S4 were previously published in Ref. [14]. For Fig. 6, new Capture-C experiments were performed using the same methods and cell lines as Ref. [14]. Full details of how the data were processed so as to compare with the simulation results are given in Additional file 2: Supplementary Methods.

### Fluorescence in situ hybridization data

Figure 3c, d and Additional file 7: Figures S5c–g show distributions of the separation of probe pairs at different locations in the *α* globin locus in mouse erythroblasts, where the *α* genes are active. Genomic locations of the probes are given in Additional file 7: Figure S5a. Probes were constructed in the pBS (pBlueScript) plasmid by subcloning regions from mouse BACRP23-469I8 and BACRP24-278E18 (obtained from CHORI, Children’s Hospital Oakland Research Institute) by *λ*-red-mediated recombination using oligonucleotide sequences shown in Additional file 16: Table S2. Recombineering was carried out mixing 50 µl of cells with 150–300 ng of purified DNA in a 0.1-cm wide cuvette using a Bio-Rad gene pulser set at 1.8 kV. Immediately after electroporation, 1 ml of SOC media (Super Optimal broth with Catabolite repression) media was added, and cells were further grown at 37 °C for 1 hour before being plated on selective agar media containing 100 µg/ml ampicillin.

In vitro cultured mouse fetal liver cells (expressing *α* and *β* globin genes) were settled on poly-l-lysine coated coverslips, fixed with 4 % paraformaldehyde in 0.25 M HEPES (4-(2-hydroxyethyl)-1-piperazineethanesulfonic acid) and permeabilized with 0.2 % Triton-X 100. FISH was performed using 7-kbp plasmid FISH probes, labeled with either Cy3-dCTP (GE Healthcare Life Sciences) or digoxygenin 11-dUTP (Roche Life Science). The genomic locations of the FISH probes are shown in Additional file 7: Figure S5a. Probes were hybridized in pairs (as in Additional file 7: Figure S5b, d–g). Following hybridization and detection using sheep anti-digoxygenin FITC (Roche Life Sciences) and rabbit anti-sheep FITC (Vector Laboratories), nuclei were imaged on a Deltavision Elite (GE Healthcare Life Sciences) using 100 × super-plan apochromat oil 1.4 N.A. objective (Olympus) with a *z*-step size of 200 nm. Images were restored by deconvolution using Huygens Professional software (Scientific Volume Imaging). Probe signal pairs were analyzed using a specifically designed Fiji algorithm that measures the 3D Euclidean distance (in microns) between thresholded signal centroids. Each measurement was adjusted to account for chromatic shift by using a displacement vector calculated from 0.1-µm Tetraspeck™ microspheres (Life Technologies) collected using the same imaging parameters as in the experiments.

We can parametrize the physical size of the chromatin beads in our simulations by fitting to the mean separation of each pair of probes as measured in the experiment. Additional file 7: Figure S5b shows a scatter plot of mean values from each pair of probes, with error bars showing the standard error in the mean; we use a linear least-squares fit weighted using the experimental error in the mean to estimate the bead diameter as 15.8 nm. Since we fit to the mean for all probe pairs, the quality of the predicted distributions can still be assessed by comparing the simulation and experiment for each individually.

### Availability of supporting data

The data sets supporting the results of this article are available in the Edinburgh DataShare repository [http://dx.doi.org/10.7488/ds/1306], including the new experimental data, simulation output data, simulation input data and scripts. Simulations were performed using the LAMMPS Molecular Dynamics Simulator [54], which is an open-source code [http://lammps.sandia.gov]. Previously published data used in the work are available at the Gene Expression Omnibus database under accession numbers GSE49460 (DNase-seq, H3K4me1 and H4K4me3 ChIP-seq for Ter119+ cells), GSE21877 (Scl/TAL1 ChIP-seq for Ter119+ cells), GSE20478 (Klf1 ChIP-seq for Ter119+ cells), GSE47492 (CTCF, GATA1 and Nfe2 ChIP-seq for Ter119+ cells), GSE47758 (Capture-C data for the *α* and *β* globin loci in Ter119+ and mES cells) and GSE67959 (Capture-C data for mitoferrin1 in Ter119+ and mES cells). Other data sets used were obtained from the ENCODE project (UCSC Accession wgEncodeEM001703 for CTCF ChIP-seq in mES cells, wgEncodeEM003417 for DNase-seq in mES cells and wgEncodeEM001681 for H3K4me1 in mES cells).

### Ethical approval

No ethical approval was required for this study.

## Additional files

Additional file 1
**Figure S1**. Chromatin is modeled as a bead-and-spring polymer; schematic diagram describing the model set-up. (PDF 69 kb)

Additional file 2
**Supplementary Methods.** Further details of the simulation scheme; full details of the ChIP-seq, DNase-seq and Capture-C data analysis (which uses software and methods from Refs. [59–63]); and full details of the clustering analysis (which uses software from [64]) and the $\mathcal {Q}$ score. (PDF 122 kb)

Additional file 3
**Figure S2**. ChIP-seq and DNase-seq data are used as an input to the model. (PDF 420 kb)

Additional file 4
**Movie 1.** Three-dimensional view of example simulated configurations of the *α* globin locus. (MP4 14,028 kb)

Additional file 5
**Figure S3**. Interactions between promoters and specific regulatory elements can be identified in each simulated conformation. (PDF 101 kb)

Additional file 6
**Figure S4**. Capture-C data confirms many of the long range chromatin interactions within the *α* globin locus that are predicted by simulations. (PDF 144 kb)

Additional file 7
**Figure S5**. Simulation results show good agreement with fluorescence in situ hybridization measurements. (PDF 94 kb)

Additional file 8
**Figure S6**. Variation of model parameters does not lead to large changes in the resulting configurations. (PDF 93 kb)

Additional file 9
**Figure S7**. ChIP-seq and DNase-seq data are used as input to a model of the *β* globin locus. (PDF 309 kb)

Additional file 10
**Figure S8**. Interactions between *β* globin promoters and specific regulatory elements can be identified in each simulated conformation. (PDF 261 kb)

Additional file 11
**Figure S9**. Simulations predict the effect of protein knock-outs on the *β* globin locus. (PDF 553 kb)

Additional file 12
**Figure S10**. ChIP-seq and DNase-seq data from mES cells can also be used as input. (PDF 136 kb)

Additional file 13
**Figure S11**. Input data are available for less well-studied loci. (PDF 415 kb)

Additional file 14
**Figure S12**. A more detailed model can explain locus folding when the R2 element is deleted. (PDF 107 kb)

Additional file 15
**Table S1**. List of all simulation parameters. (PDF 93 kb)

Additional file 16
**Table S2**. Oligonucleotide sequences for FISH probes. (PDF 68 kb)
