# Supplementary material for: Predicting the three-dimensional folding of cis-regulatory regions in mammalian genomes using bioinformatic data and polymer models
Source: Genome Biol. 2016 Mar 31;17:59. doi: 10.1186/s13059-016-0909-0 (PMC4815170; doi:10.1186/s13059-016-0909-0)
Supplement: Additional file 1 — Figure S1. Chromatin is modeled as a bead-and-spring polymer; schematic diagram describing the model set-up. (PDF 69 kb) [file 13059_2016_909_MOESM1_ESM.pdf]

Additional Files

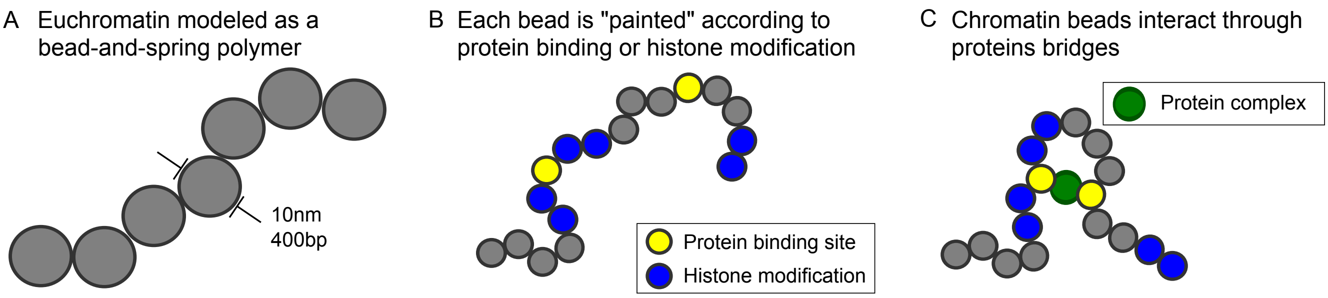

Additional file 1: Figure S1: **Chromatin is modelled as a bead-and-spring polymer.** (A) Beads represent a region of chromatin containing 400 bp of DNA, approximately two nucleosomes. This coarse graining sets the resolution of our simulations, but does not specify a particular structure for the chromatin fibre; the physical size of the bead is not specified, but fitting results to FISH measurements (see Methods and Additional file 7: Figure S5) suggests a bead diameter of 16 nm. We set the persistence length (i.e. length over which the polymer behaves like a stiff rod, and a measure of the stiffness of the polymer) to 4 bead diameters (64 nm), a reasonable choice for euchromatin (1,55). Changing this parameter does not significantly affect our results (see Additional file 8: Figure S6). (B) and (C) Experimental data such as ChIP-seq or DNase-seq is used to specify which beads can be bound by the protein complexes in our simulations.
