# Supplementary material for: Predicting the three-dimensional folding of cis-regulatory regions in mammalian genomes using bioinformatic data and polymer models
Source: Genome Biol. 2016 Mar 31;17:59. doi: 10.1186/s13059-016-0909-0 (PMC4815170; doi:10.1186/s13059-016-0909-0)
Supplement: Additional file 2 — Supplementary Methods. Further details of the simulation scheme; full details of the ChIP-seq, DNase-seq and Capture-C data analysis (which uses software and methods from Refs. [59–63]); and full details of the clustering analysis (which uses software from [64]) and the $\mathcal {Q}$Q score. (PDF 122 kb) [file 13059_2016_909_MOESM2_ESM.pdf]

# Additional file 2: Supporting Methods

**Langevin dynamics simulations.** Chromatin regions and protein complexes are represented by beads, and the position of the $i$th bead in the system evolves according to the Langevin equation

$$m_i \frac{d^2\mathbf{r}_i}{dt^2} = -\nabla U_i - \gamma_i \frac{d\mathbf{r}_i}{dt} + \sqrt{2k_BT\gamma_i}\eta_i(t), \quad \text{[S1]}$$

where $\mathbf{r}_i$ is the position of bead $i$ with mass $m_i$, $\gamma_i$ is the friction due to an implied solvent, and $\eta_i$ is a vector representing random uncorrelated noise such that

$$\langle \eta_\alpha(t) \rangle = 0 \text{ and } \langle \eta_\alpha(t)\eta_\beta(t')\rangle = \delta_{\alpha\beta}\delta(t-t'). \quad \text{[S2]}$$

The noise is scaled by the energy of the system, given by the Boltzmann factor $k_B$ multiplied by the temperature of the system $T$, taken to be 310 K for a cell. The potential $U_i$ is a sum of interactions between bead $i$ and all other beads, and we use phenomenological interaction potentials as described below. For simplicity we assume that all beads in the system have the same mass $m_i = m$. Equation (S1) is solved in LAMMPS using a standard Velocity-Verlet algorithm.

For the chromatin fibre the $i$th bead in the chain is connected to the $i+1$th with a with a finitely extensible non-linear elastic (FENE) spring given by the potential

$$U_{\text{FENE}}(r_{i,i+1}) = U_{\text{WCA}}(r_{i,i+1}) - \frac{K_{\text{FENE}}R_0^2}{2} \log\left[1 - \left(\frac{r_{i,i+1}}{R_0}\right)^2\right], \quad \text{[S3]}$$

where $r_{i,i+1} = |\mathbf{r}_i - \mathbf{r}_{i+1}|$ is the separation of the beads, and the first term is the Weeks-Chandler-Andersen (WCA) potential

$$\frac{U_{\text{WCA}}(r_{ij})}{k_BT} = \begin{cases} 4\left[\left(\frac{d_{ij}}{r_{ij}}\right)^{12} - \left(\frac{d_{ij}}{r_{ij}}\right)^{6}\right] + 1, & r_{ij} < 2^{1/6}d_{ij} \\ 0, & \text{otherwise,} \end{cases} \quad \text{[S4]}$$

which represents a hard steric interaction which prevents adjacent beads from overlapping; here $d_{ij}$ is the mean of the diameters of beads $i$ and $j$. The diameter of the chromatin beads is a natural length scale with which to parametrize the system; we denote this $\sigma$, and use this to define all other length scales. The second term in Eq. (S3) gives the maximum extension of the bond, $R_0$; throughout we use $R_0 = 1.6\ \sigma$, and set the bond energy $K_{\text{FENE}} = 30\ k_BT$. The bending rigidity of the polymer is introduced via a Kratky-Porod potential for every three adjacent DNA beads

$$U_{\text{BEND}}(\theta) = K_{\text{BEND}}\left[1 - \cos(\theta)\right], \quad \text{[S5]}$$

where $\theta$ is the angle between the three beads as give by

$$\cos(\theta) = [\mathbf{r}_i - \mathbf{r}_{i-1}] \cdot [\mathbf{r}_{i+1} - \mathbf{r}_i], \quad \text{[S6]}$$

and $K_{\text{BEND}}$ is the bending energy. The persistence length in units of $\sigma$ is given by $l_p = K_{\text{BEND}}/k_BT$. Finally, steric interactions between non-adjacent DNA beads are also given by the WCA potential [Eq. (S4)].

Each protein complex is represented by a single bead and the WCA potential is used to give a steric interaction between these. Chromatin beads are labelled as binding or not-binding for each protein species according to the input data (see section on ChIP-seq and DNase-seq data analysis below). For the interaction between proteins and the chromatin beads labelled as binding, we use a shifted, truncated Lennard-Jones potential

$$U_{\text{LJcut}}(r_{ij}) = \begin{cases} U_{\text{LJ0}}(r_{ij}) - U_{\text{LJ0}}(r_{\text{cut}}) & r_{ij} < r_{\text{cut}}, \\ 0 & \text{otherwise,} \end{cases} \quad \text{[S7]}$$

with

$$U_{\text{LJ0}}(r) = 4\varepsilon'\left[\left(\frac{d_{ij}}{r}\right)^{12} - \left(\frac{d_{ij}}{r}\right)^{6}\right],$$

where $r_{\text{cut}}$ is a cut off distance, and $r_{ij}$ and $d_{ij}$ are the separation and mean diameter of the two beads respectively. This leads to an attraction between a protein and a chromatin bead if their centres are within a distance $r_{\text{cut}}$. Here $\varepsilon'$ is an energy scale, but due to the second term in Eq. (S7) this is not the same as the minimum of the potential, which for clarity we denote $\varepsilon$ (and we refer this to as the interaction energy). For simplicity we set the diameter of the protein complexes equal to that of the chromatin beads, $d_{ij} = \sigma$, and set $r_{\text{cut}} = 1.4\ \sigma$.

The polymer is initialized as a random walk, and the dynamics are first evolved in the absence of protein interactions in order to generate an equilibrium coil conformation. Interactions with the protein complexes are then switched on, and the dynamics are evolved until a new equilibrium conformation is obtained. The length scale $\sigma$, mass $m$ and energy scale $k_BT$ give rise to a natural simulation time unit $\tau_{\text{LJ}} = \sqrt{\sigma^2 m/k_BT}$, and Eq. (S1) is integrated with a constant time step $\Delta t = 0.01\tau_{\text{LJ}}$, for a total of at least $8 \times 10^6$ time steps. Each simulation is repeated at least 500 times using a different initial conformation and random noise, resulting in an ensemble of conformations. Two chromatin beads are said to be interacting if their separation is less than 2.75 bead diameters; counting the proportion of conformations in which a given pair of beads is interacting gives an approximation of the probability that those beads interact.

So far the system has been described in units $\sigma$, $m$, and $k_BT$. In order to map these simulation units to real ones we must recognise that there are two further important time scales in the system, namely the inertial time $\tau_{\text{in}} = m/\gamma_i$ (from Eq. (S1) this is the time over which a bead loses information about its velocity), and the Brownian time $\tau_{\text{B}} = \sigma^2/D_i$ (the time it takes for a bead to diffuse across its own diameter $\sigma$). Here $D_i$ is the diffusion constant for bead $i$, given

through the Einstein relation by $D_i = k_B T/\gamma_i$; if we make the approximation that a chromatin bead will diffuse like a sphere we can then use Stokes' Law, where $\gamma_i = 3\pi\nu d_i$, with $\nu$ the viscosity of the fluid, and $d_i$ the diameter of bead $i$. Taking realistic values for the length, mass and viscosity one finds that $\tau_{\rm in} \ll \tau_{\rm LJ} \ll \tau_{\rm B}$, with the times separated by several orders of magnitude. For numerical stability we must choose the time step $\Delta t$ smaller than all of these times, and we wish to study phenomena which will occur on times of the order $\tau_{\rm B}$; this means that using real values for all parameters would lead to infeasibly long simulation run times. Instead we make an approximation by setting $m = k_B T = \sigma = 1$, and $\gamma_i = 2$, and map to real time scales through the Brownian time $\tau_{\rm B}$; although this means that beads in our simulation have more inertia than in reality, this does not effect our results, which are taken once the polymer has reached an equilibrium conformation. Taking the diameter of the chromatin beads to be 15.8 nm, and assuming a viscosity of 10 cP for the nucleoplasm gives $\tau_{\rm B} \approx 87$ $\mu$s, meaning that a simulation time unit is $\approx 43.5$ $\mu$s. Each simulation run therefore represents approximately 7 s of real time.

**ChIP-seq and DNase-seq data analysis.** As an input to the model we use ChIP-seq and DNase-seq data (previously published in Refs. (14,50,56-58) as indicated in the captions for Additional files 3, 9 and 12: Figures S2, S7 and S10) to identify protein binding sites in the chromosome region of interest. For protein binding, ChIP-seq reads are aligned to the mouse reference genome build mm9 using the Bowtie2 software (59); duplicate reads are removed, and pile-ups are generated using the BedTools package (60). Binding sites are identified using the macs2 peak calling software (61) using a control data set where available; peaks which have a normalised $p$-value $< 0.001$, and which have a fold-change higher than a threshold are retained. DNase-seq reads are similarly aligned to the mm9 genome using Bowtie2, but peaks are identified using the PeaKDEck software (which uses a peak finding algorithm calibrated specifically for DNase-seq data (62)). As detailed in the main text, we simplify our model by assuming that DNase hypersensitive sites indicate the positions of transcription factor binding sites. For histone modifications, we also align reads using Bowtie2; since these modifications can be found across wide regions, rather than identifying peaks we instead find regions where the pile-up of reads exceeds a threshold.

In order to incorporate the data into the simulations, the locus of interest is divided into regions corresponding to each bead in our model chromatin fibre. Beads are then labelled according to any peak or histone modification which overlaps with the region; for simplicity we only label beads a binding or not (or as having a histone modification or not), and do not incorporate peak intensities into the model.

**Cluster Analysis.** In order to assess the similarity between the conformations generated in each set of simulations we perform a cluster analysis. First we calculate the generalised "distance" between all pairs of conformations; then a dendrogram is generated using the standard hierarchical clustering algorithm in the MATLAB software (64), with an average linkage criterion.

A standard way to measure the distance between two polymer conformations is to consider the mean squared difference between separations of pairs of beads in each; however since our polymer consists of regions which bind proteins and unstructured regions, this does not perform well (the unstructured regions dominate in the mean, and no clear clusters are found). Instead we use a distance $\Gamma(C,C')$ between conformations $C$ and $C'$ which ignores the unstructured regions, defined as

$$\Gamma(C,C') = \frac{1}{(n(n-1))/2}\sum_{i\neq j}[1-\delta_{s_{ij}^C,s_{ij}^{C'}}](r_{ij}^C - r_{ij}^{C'})^2, \quad \text{[S8]}$$

where $r_{ij}^C$ is the separation of beads $i$ and $j$ in conformation $C$. The Kronecker $\delta$-function is defined such that $\delta_{a,b} = 1$ if $a = b$ and 0 otherwise, with $s_{ij}^C = 1$ if beads $i$ and $j$ are interacting in conformation $C$ and 0 otherwise (an interaction is defined as having separation less than 2.75 bead diameters). Thus the only contributions to the mean are from beads which are interacting in one conformation but not in the other; further limiting the analysis to consider only the chromatin beads within the most structured region of the locus (indicated by green bars in Figures 1C and 4C) results in a series of well defined clusters (Figures 2 and 4D).

**Capture-C data.** In order to test the predictions of the model we compared simulation results with Capture-C data; for the $\alpha$ and $\beta$ globin loci data were from Ref. (14), whereas data for the mitoferrin locus in Figure 6 were from new experiments performed according to the method in that reference. In these experiments a set of oligonucloetide capture "targets" is designed, a 3C library is obtained using a frequently cutting restriction enzyme (Dpn II, cutting at GATA), and SureSelect oligonucleotide capture is followed by Hi-seq paired-end sequencing. The resulting reads then undergo *in silico* DpnII digestion (producing a set of fragments for each read), and the fragments are aligned to the mouse mm9 reference genome as single-end reads using the Bowtie software (63). Identical sets of read fragments are assumed to be PCR artefacts, and are removed (14); read sets which contain a targeted restriction fragment and a reporter fragment are retained. Data are then smoothed by counting interactions within 800 bp windows centred on genomic positions separated by 400 bp steps, giving an interaction profile for each target (black lines with grey shading in Figures 3A,B, 4E,F and 5C,F, and Additional Files 6 and 14: Figures S4 and S12). Since the efficiency of capture of each target is unknown, the obtained profiles show *relative* interaction strength, and profiles from different targets cannot be compared quantitatively. Reads showing interactions between targeted regions could have been captured from either target, so these reads are not quantitative and must be removed; these regions are indicated by black blocks in Figures 3A,B, 4E,F and 5C,F, and Additional Files 6 and 14: Figures S4 and S12.

To compare Capture-C data with our simulated interaction profiles we first identify the simulation beads that correspond to each of the targeted regions. From the ensemble of simulated conformations we find the probability that that any chromatin bead within the target region is interacting (separation less than 2.75 bead diameters) with each other bead (the probability is approximated by $n/N$ when there is an interaction in $n$ conformations in a set of $N$). Since the Capture-C experiment only gives relative interaction profiles, to plot the data on the same axis as simulations we must scale it by a factor $\gamma$ which we find via a least squares fit. After removing interactions between targets from both the simulation and experimental data sets, we use cubic spline interpolation to obtain points at the same genomic locations for each data set; in a plot with simulation and experimental values on the axes, $\gamma$ is this slope of a linear fit which goes through zero.

**Quantitative comparison with experimental data - the $\mathscr{Q}$ score.** In order to quantitatively compare our simulations with data from Capture-C experiments we define a score, denoted $\mathscr{Q}$ which takes a value between 0 and 1 depending on the overlap between chromatin interaction peaks which are predicted by simulations, and those observed in experiments ($\mathscr{Q} = 1$ denoting perfect overlap). For a data set for a given capture target we first normalise by dividing by the number of interactions in the vicinity of the target; we then scale all of the experimental data so that it best fits the simulation. A sliding averaging window is used to smooth both the simulation and experimental data, before applying a peak finding algorithm to identify interactions (the "findpeaks" function in the MATLAB software (64)). We use the peak positions and widths (but not heights) to test whether peaks in each data set overlap, and calculate a value

$$q_i = \frac{n_{\mathrm{se}} + n_{\mathrm{es}}}{n_{\mathrm{s}} + n_{\mathrm{e}}}, \qquad \text{[S9]}$$

where $n_{\mathrm{s}}$ and $n_{\mathrm{e}}$ are the number of peaks found in the simulation and experimental data respectively, $n_{\mathrm{se}}$ is the number of peaks in the simulation data which overlap with one or more peaks in the experimental data, and $n_{\mathrm{es}}$ is the number of peaks in the experimental data which overlap with one or more peaks in the simulation data. It is possible for $n_{\mathrm{se}}$ and $n_{\mathrm{es}}$ to differ if, for example, two adjacent peaks in the simulation overlap a single broader peak in the experiment. Since from a single simulation and experiment we compare data from each capture target separately, we take an average to find an overall score $\mathscr{Q} = \sum_i q_i$, where $q_i$ is the score for the $i$th capture target. Note that since the experimental data is always scaled so as to best fit the simulation (necessary since the Capture-C signal is in units of numbers of reads, and we do not know the proportionality constant which relates this to the probability of two regions interacting), simulations always score reasonably well, and defining a measure of their quality is very difficult. To set the scale, we compare with a simulation where the bead colourings are shuffled randomly.

In Additional file 8: Figure S6 we compare $\mathscr{Q}$ scores for a number of different simulation models. To generate the "shuffled" chromatin fibre, the bead colourings are shuffled subject to two constraints: first, in order to preserve e.g. the pattern of histone methylation around the DHS or CTCF sites, we keep groups of 10 adjacent beads (4 kbp) together, and second, so that there are some interactions to compare we preserve the bead colouring at the targets used in the experiment (if a protein binding site were shuffled away from a target, then there would be very little long range interaction with that region).

Quantifying the difference between two different sets of simulations is more straightforward, since no scaling is required. We define

$$\chi^2(A,B) = \frac{1}{(n(n-7))} \sum_{|i-j|>6} [P_A(i,j) - P_B(i,j)]^2, \qquad \text{[S10]}$$

where $P_A(i,j)$ is the probability that chromatin beads $i$ and $j$ are in contact in set of simulations $A$ (i.e. the values shown in contact maps), and the sum runs over all pairs of beads which have a linear separation greater than 6 (this means the diagonal in contact maps are not included in the comparison). $\chi^2$ gives the difference between two contact maps, i.e. the larger its value the more different the two sets of experiments.
