# Supplementary material for: Predicting the three-dimensional folding of cis-regulatory regions in mammalian genomes using bioinformatic data and polymer models
Source: Genome Biol. 2016 Mar 31;17:59. doi: 10.1186/s13059-016-0909-0 (PMC4815170; doi:10.1186/s13059-016-0909-0)
Supplement: Additional file 5 — Figure S3. Interactions between promoters and specific regulatory elements can be identified in each simulated conformation. (PDF 101 kb) [file 13059_2016_909_MOESM5_ESM.pdf]

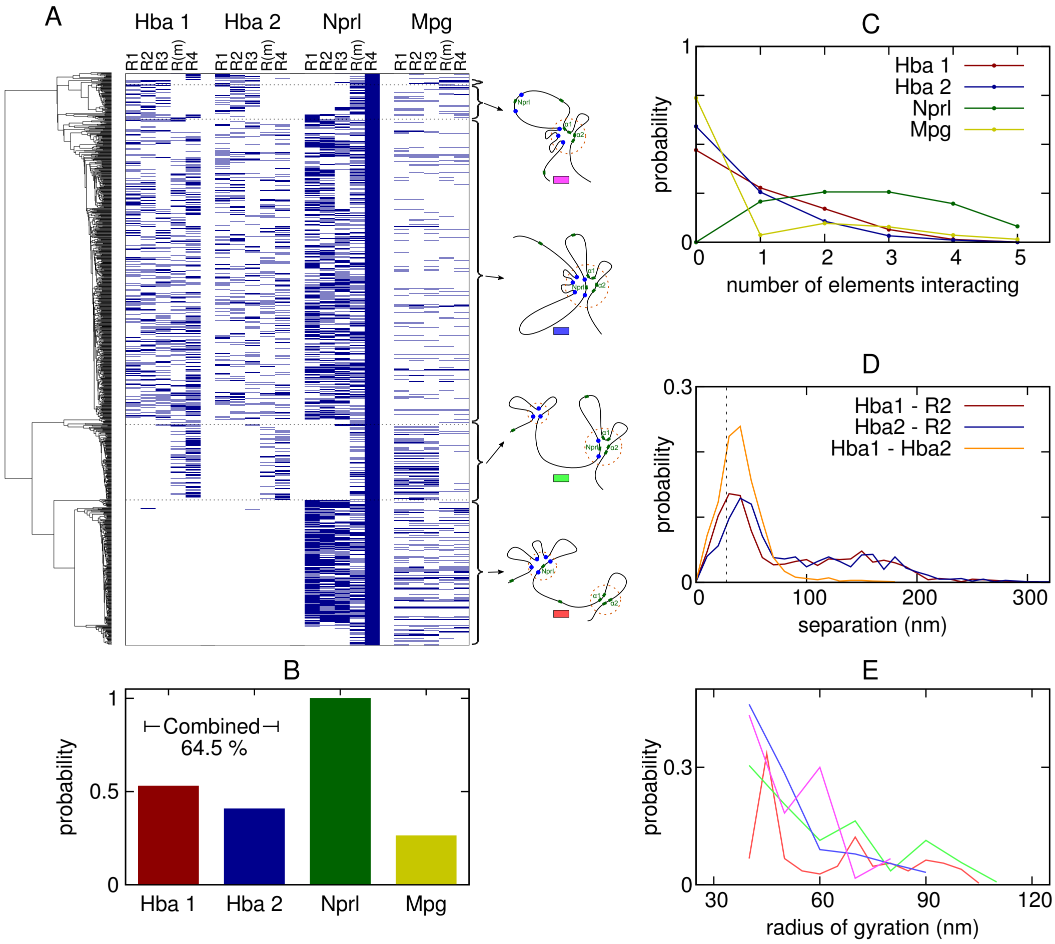

Additional file 5: Figure S3: **Interactions between promoters and specific regulatory elements can be identified in each simulated conformation.** (A) Plot showing details of which promoters are interacting with each of the five known regulatory elements in the same set of simulations as presented in Figures 1-3. Each horizontal row represents a single simulated conformation, with a blue mark indicating there is an interaction with the element (an interaction is defined as any chromatin bead lying within the promoter being within 2.75 bead diameters of any chromatin bead within the regulatory element). The grouping of different types of structure according to the clustering analysis is indicated to the left. (B) Plot showing in what proportion of conformations each of the promoters is interacting with one or more of the regulatory elements. The proportion of conformations in which either one of the α globin promoters is interacting with any of the elements is also indicated; one would expect this to represent the proportion of conformation in which α globin is being transcribed. (C) Histograms showing the distribution of the number of elements with which each promoter simultaneously interacts in a given conformation. (D) Histograms showing the distributions of the 3D separation between each of the two α globin gene promoters, and the R2 regulatory element. Also shown is the distribution for the separation between the two promoters. The dashed line indicates the distance below which two chromatin beads are deemed to be interacting. (E) Histograms showing the distribution of the radius of gyration of the locus in the conformations adopting each structure identified by the clustering analysis. The colour correspond to the different structures as shown in the schematics in panel A.
