# Supplementary material for: Predicting the three-dimensional folding of cis-regulatory regions in mammalian genomes using bioinformatic data and polymer models
Source: Genome Biol. 2016 Mar 31;17:59. doi: 10.1186/s13059-016-0909-0 (PMC4815170; doi:10.1186/s13059-016-0909-0)
Supplement: Additional file 6 — Figure S4. Capture-C data confirms many of the long range chromatin interactions within the α globin locus that are predicted by simulations. (PDF 144 kb) [file 13059_2016_909_MOESM6_ESM.pdf]

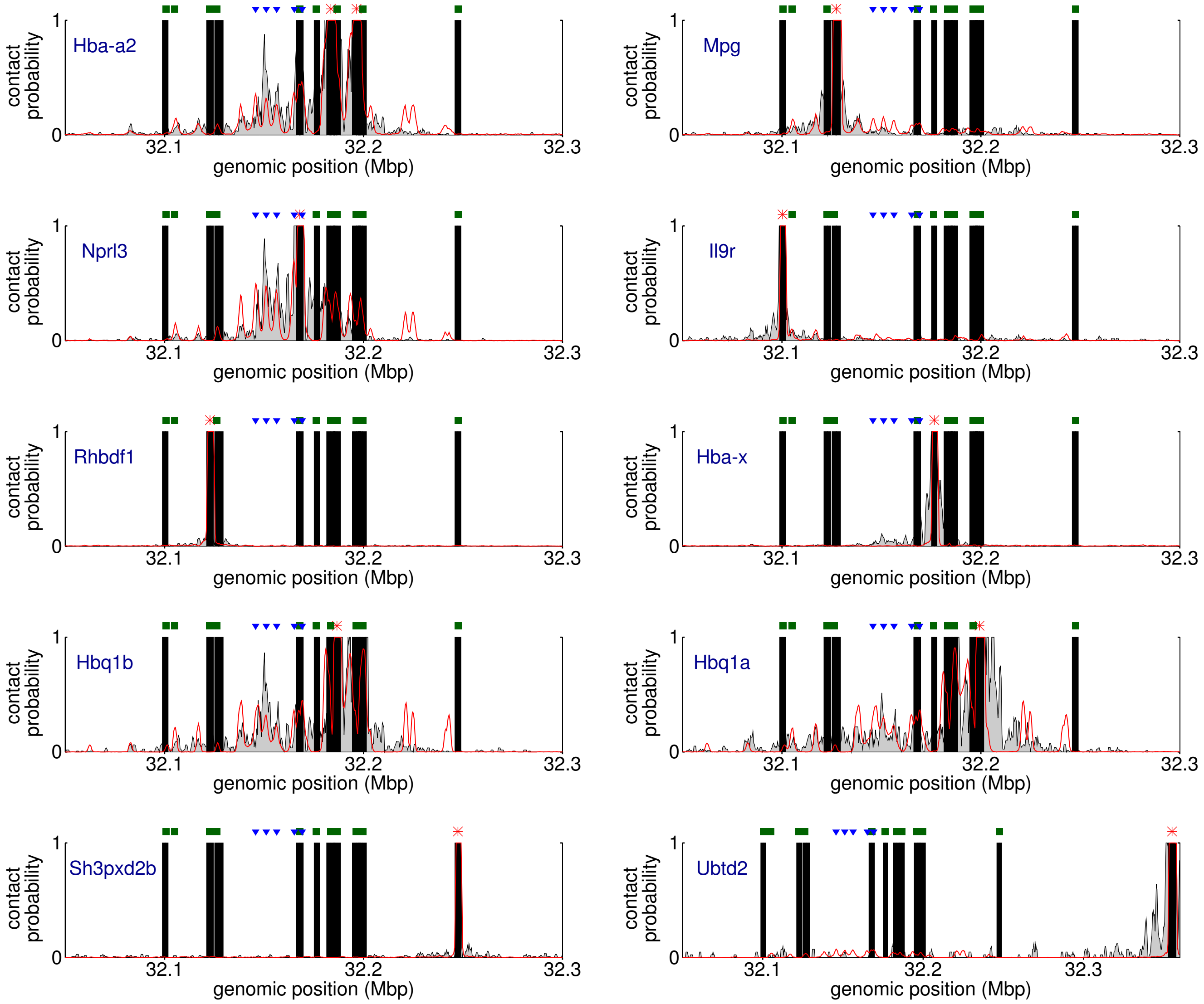

Additional file 6: Figure S4: **Capture-C data confirms many of the long range chromatin interactions within the α globin locus which are predicted by simulations.** Plots showing results from the simulations described in Figures 1-3 alongside data from Capture-C experiments. Capture-C results are from Ref. (14), and plots are shown for each capture probe from that data set; these are at the locations of all of the promoters within the region simulated. Red lines show simulation results, black shaded curves the experimental data, and black bars indicate regions where no experimental data is available. The experimental data are scaled as described in Additional file 2: Supplementary Methods. The positions of the known regulatory elements and other promoters are indicated at the top of each plot with blue and green symbols respectively, and the red stars indicate the position of the Capture-C probe for each plot.
