# Supplementary material for: Predicting the three-dimensional folding of cis-regulatory regions in mammalian genomes using bioinformatic data and polymer models
Source: Genome Biol. 2016 Mar 31;17:59. doi: 10.1186/s13059-016-0909-0 (PMC4815170; doi:10.1186/s13059-016-0909-0)
Supplement: Additional file 8 — Figure S6. Variation of model parameters does not lead to large changes in the resulting configurations. (PDF 93 kb) [file 13059_2016_909_MOESM8_ESM.pdf]

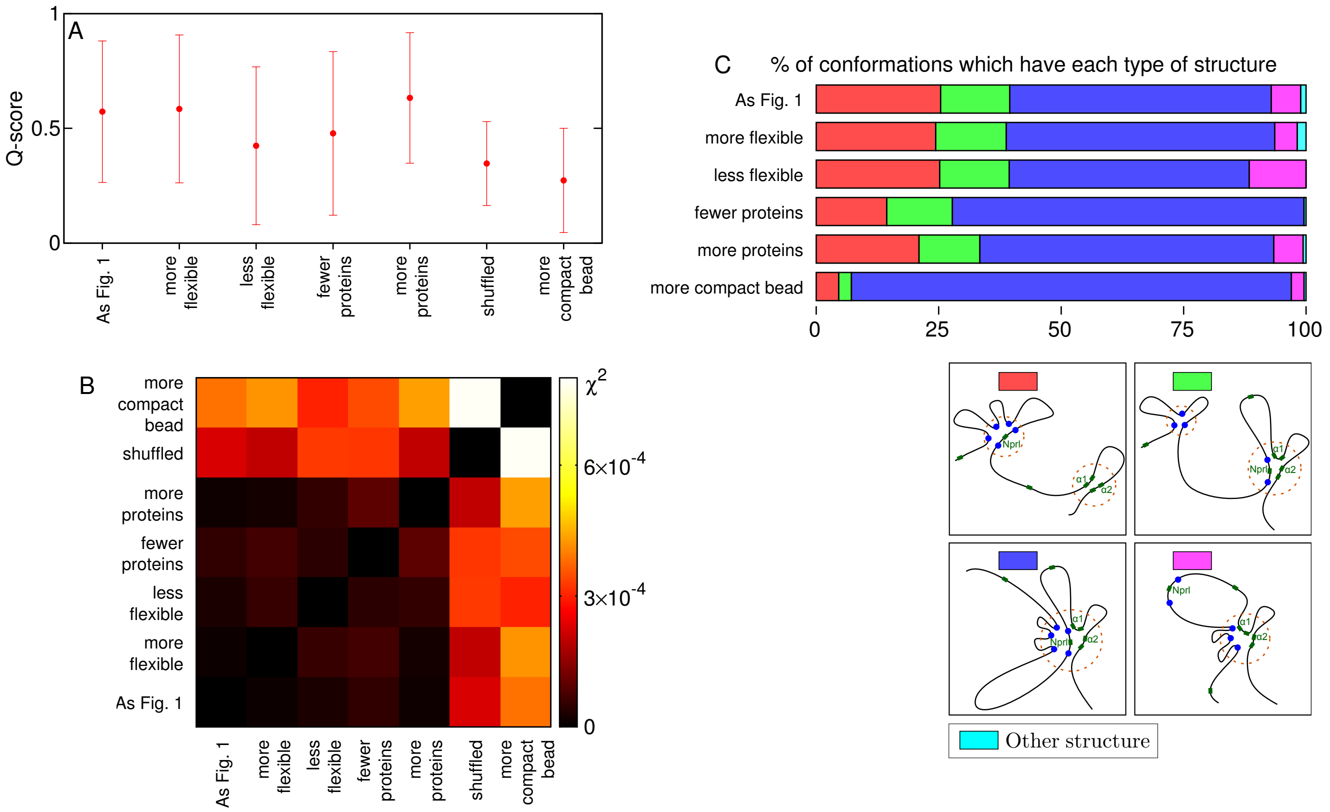

Additional file 8: Figure S6: **Variation of model parameters does not lead to large changes in the resulting configurations.** (A) Plot showing how the $\mathcal{Q}$ score, which quantifies the agreement between the simulation and experimental chromosome interactions, varies with different model parameters (see Additional file 2: Supplementary Methods). Points show the mean over the set of targets captured in the experiment, and error bars show the error in this mean. The flexibility of the polymer was varied (making is less flexible by increasing the persistence length from 4 bead diameters to 8 bead diameters, or making it more flexible by decreasing the persistence length to 2 bead diameters); the number of protein complexes was varied, either decreasing from 20 to 10 copies of each species, or increasing to 30 of each species. We also considered a simulation where the colouring of each bead was randomly shuffled (see Additional file 2: Supplementary Methods). Finally, we reduced the resolution of the model by increasing the amount of chromatin represented by each bead from 400 bp to 600 bp. (B) Plot showing how the contact maps differ between each set of experiments, measured by χ^2 (see Additional file 2: Supplementary Methods). (C) Plot showing the proportion of conformations found to be forming the different structures identified by the clustering analysis. Schematics of these structures are shown below the plot.
