# Supplementary material for: Predicting the three-dimensional folding of cis-regulatory regions in mammalian genomes using bioinformatic data and polymer models
Source: Genome Biol. 2016 Mar 31;17:59. doi: 10.1186/s13059-016-0909-0 (PMC4815170; doi:10.1186/s13059-016-0909-0)
Supplement: Additional file 9 — Figure S7. ChIP-seq and DNase-seq data are used as input to a model of the β globin locus. (PDF 309 kb) [file 13059_2016_909_MOESM9_ESM.pdf]

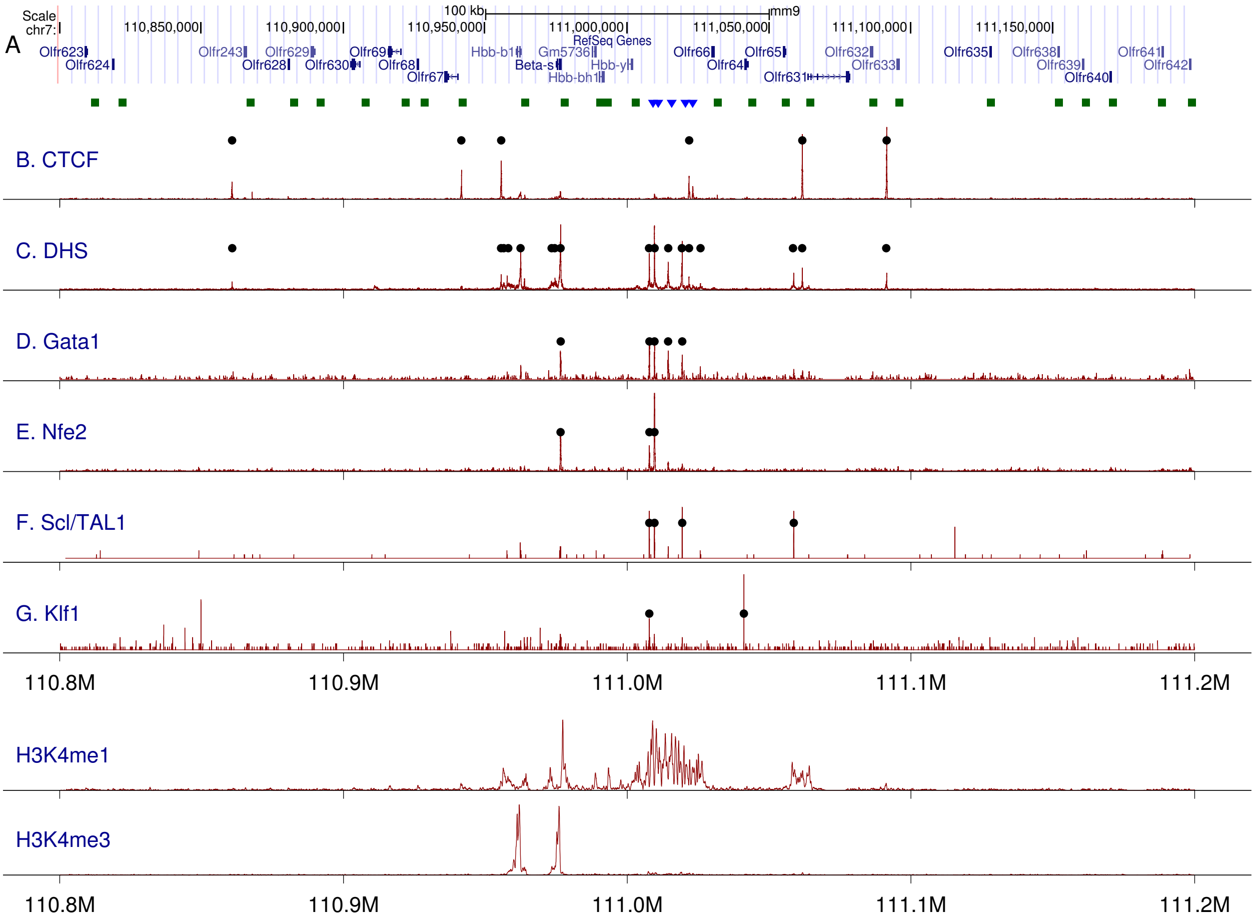

Additional file 9: Figure S7: **ChIP-seq and DNase-seq data are used as an input to a model of the β globin locus.** (A) Genome browser view of genes in a 400 kbp region of mouse chromosome 7 surrounding the β globin locus which is treated in our simulations. Symbols below the browser indicate the positions of the known regulatory elements within the LCR (blue triangles) and the gene promoters (green squares). (B) ChIP-seq data for CTCF binding across the same region from mouse erythroid (Ter119^{+}) cells. Red lines show the pile-up of reads, and black points indicate the positions of binding sites identified by peak-calling (see Additional file 2: Supplementary Methods). Data from Ref. (14). (C) Similar plot showing DNase-seq data from the same cell type, identifying the positions of DNase-1 hypersensitive sites (DHS). Data from Ref. (56). (D)-(G) Plots showing ChIP-seq data, again from the same cell type, for four TFs thought to be key players in globin regulation. Data from Ref. (14) (GATA1 and NFe2), Ref. (50) (Scl/Tal1), and Ref. (57) (Klf1). (H)-(I) ChIP-seq data showing relevant histone modifications: monomethylation and trimethylation of H3K4 (associated with enhances and promoters respectively). Data from Ref. (56). All plots are aligned according to the horizontal axis.
