# Supplementary material for: Predicting the three-dimensional folding of cis-regulatory regions in mammalian genomes using bioinformatic data and polymer models
Source: Genome Biol. 2016 Mar 31;17:59. doi: 10.1186/s13059-016-0909-0 (PMC4815170; doi:10.1186/s13059-016-0909-0)
Supplement: Additional file 10 — Figure S8. Interactions between β globin promoters and specific regulatory elements can be identified in each simulated conformation. (PDF 261 kb) [file 13059_2016_909_MOESM10_ESM.pdf]

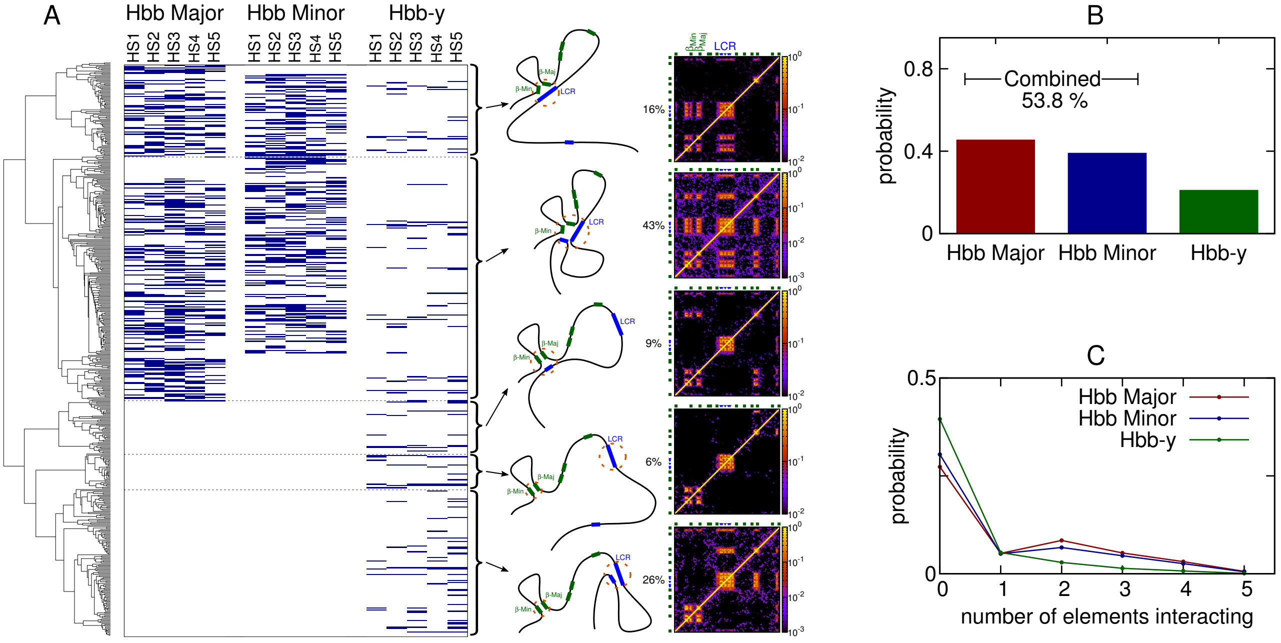

Additional file 10: Figure S8: **Interactions between β globin promoters and specific regulatory elements can be identified in each simulated conformation.** (A) Plot showing details of which promoters are interacting with the known regulatory elements within the LCR from the same set of simulations as presented in Figure 4. Each horizontal row represents a single simulated conformation, with a blue mark indicating there is an interaction with the element (an interaction is defined as any chromatin bead lying within the promoter being within 2.75 bead diameters of any chromatin bead within the regulatory element). The grouping of different types of structure according to the clustering analysis is indicated to the left; schematics and an individual contact map for each group are shown. (B) Plot showing in what proportion of conformations each of the promoters is interacting with one or more of the regulatory elements. The proportion of conformations in which either one of the β globin promoters is interacting with any of the elements is also indicated. (C) Histograms showing the distribution of the number of elements with which each promoter simultaneously interacts in a given conformation.
