# Supplementary material for: Predicting the three-dimensional folding of cis-regulatory regions in mammalian genomes using bioinformatic data and polymer models
Source: Genome Biol. 2016 Mar 31;17:59. doi: 10.1186/s13059-016-0909-0 (PMC4815170; doi:10.1186/s13059-016-0909-0)
Supplement: Additional file 11 — Figure S9. Simulations predict the effect of protein knock-outs on the β globin locus. (PDF 553 kb) [file 13059_2016_909_MOESM11_ESM.pdf]

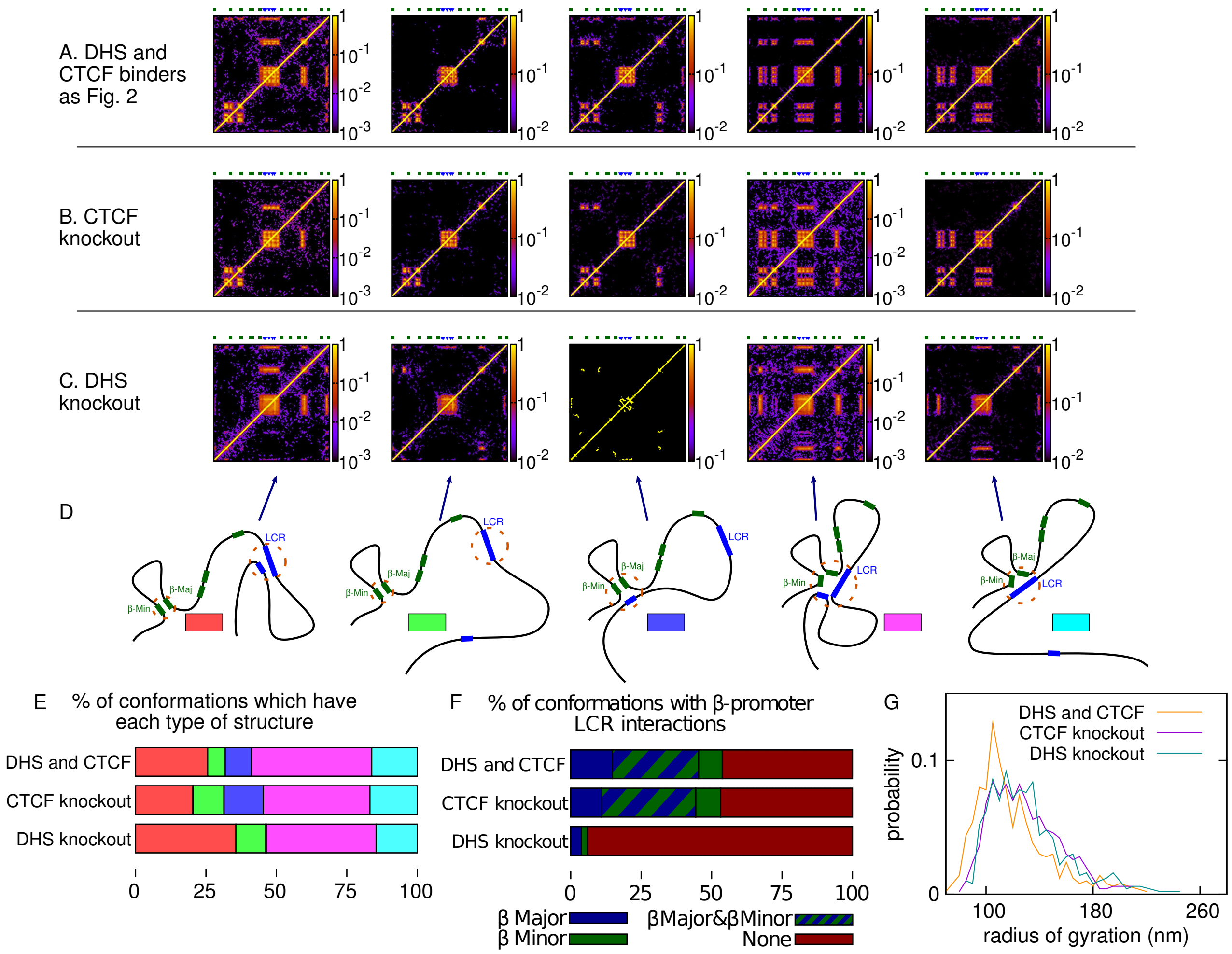

Additional file 11: Figure S9: **Simulations predict the effect of protein knock-outs on the β globin locus.** Plots showing the effect of a CTCF knock-out, and a "DHS knockout" (equivalent to knocking out all protein complexes involved in looping the β globin locus *except* CTCF). (A)-(C) Contact maps showing the interactions between different chromosomal locations for conformations within each group identified by clustering analysis. Maps from three sets of simulations are shown. (D) Schematics showing the structure of the locus within each group. (E) Plot showing the percentage of conformations which belong to each group identified by the clustering analysis. The colour key is given in D. (F) Plot showing in what percentage of conformations the two β globin gene promoters are interacting with one or more of the regulatory elements within the LCR. (G) Plot showing the distribution of the radius of gyration of the locus across the simulated conformations. The radius of gyration is defined as given in the caption to Figure 7.
