# Supplementary material for: Predicting the three-dimensional folding of cis-regulatory regions in mammalian genomes using bioinformatic data and polymer models
Source: Genome Biol. 2016 Mar 31;17:59. doi: 10.1186/s13059-016-0909-0 (PMC4815170; doi:10.1186/s13059-016-0909-0)
Supplement: Additional file 12 — Figure S10. ChIP-seq and DNase-seq data from mES cells can also be used as input. (PDF 136 kb) [file 13059_2016_909_MOESM12_ESM.pdf]

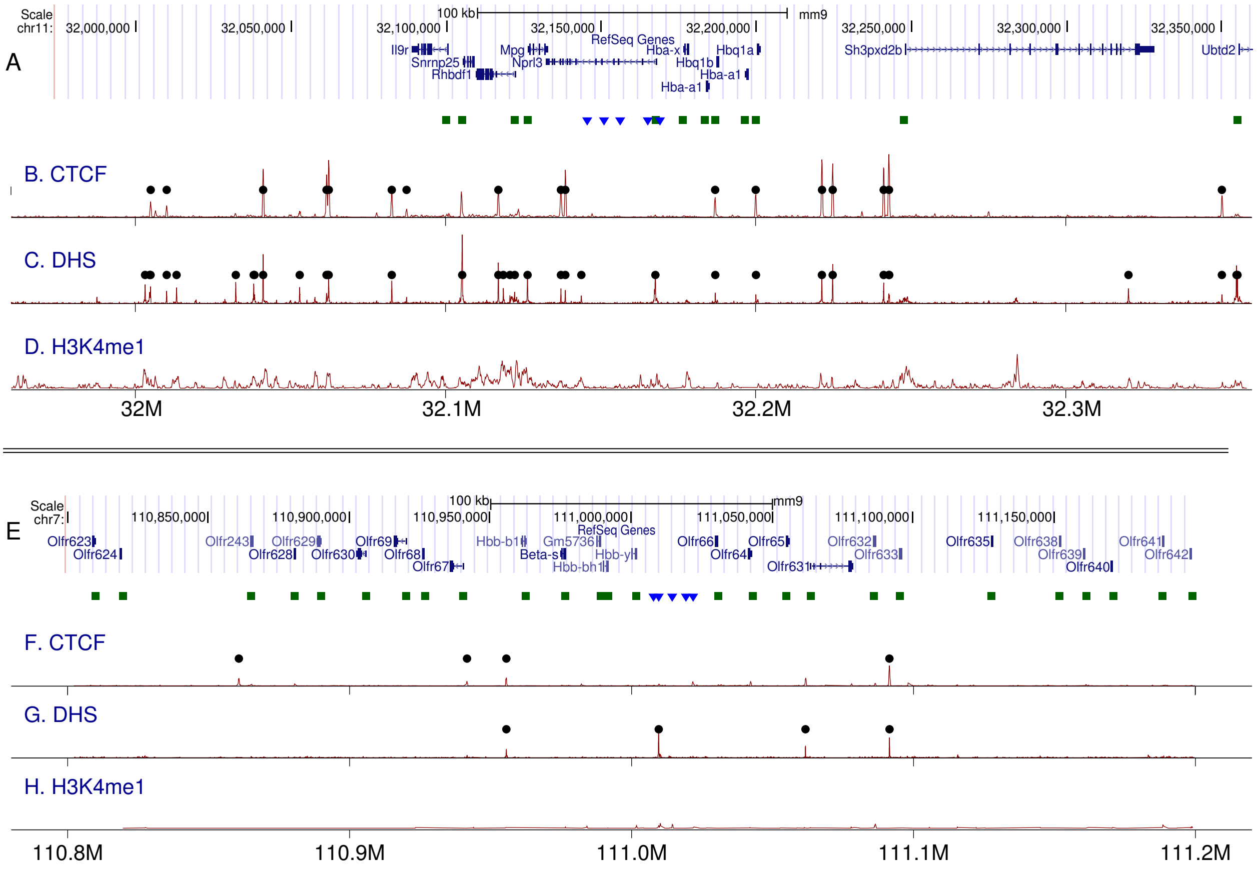

Additional file 12: Figure S10: **ChIP-seq and DNase-seq data from mES cells can also be used as an input.** (A) Browser view of the α globin locus. (B-D) ChIP-seq and DNase-seq data for mouse ES cells across the same region. Red lines show the pile-up of reads, and black points indicate the positions of binding sites identified by peak-calling. Data from the ENCODE project (58). (E) Browser view of the β globin locus. (F-H) ChIP-seq and DNase-seq data for mouse ES cells across the same region. Data from the ENCODE project (58).
