# Supplementary material for: Predicting the three-dimensional folding of cis-regulatory regions in mammalian genomes using bioinformatic data and polymer models
Source: Genome Biol. 2016 Mar 31;17:59. doi: 10.1186/s13059-016-0909-0 (PMC4815170; doi:10.1186/s13059-016-0909-0)
Supplement: Additional file 13 — Figure S11. Input data are available for less well-studied loci. (PDF 415 kb) [file 13059_2016_909_MOESM13_ESM.pdf]

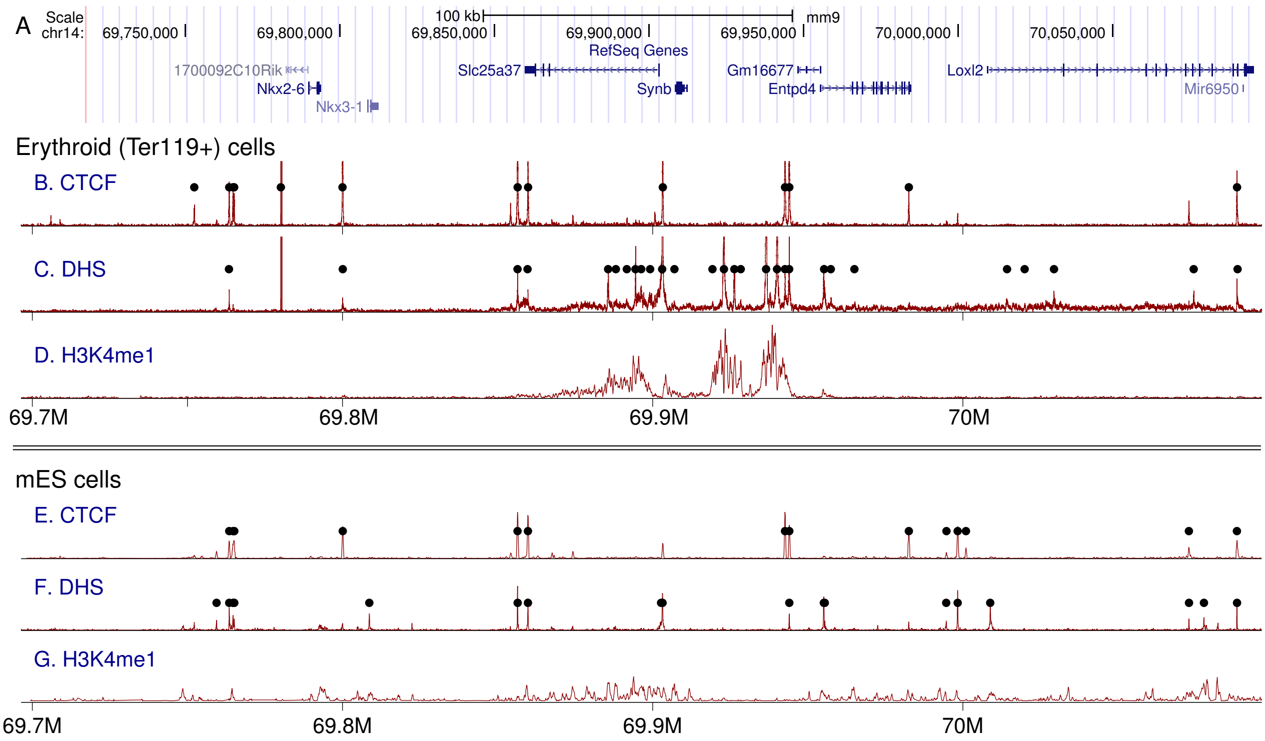


**Additional file 13: Figure S11**: **Input data is available for less well studied loci.** (A) Browser view of the *Slc25a37* (mitoferrin) locus. (B-D) ChIP-seq and DNase-seq data for mouse erythroid (Ter119+) cells across the same region. Red lines show the pile-up of reads, and black points indicate the positions of binding sites identified by peak-calling. CTCF data is from Ref. (14); DNase and histone modification data is from Ref. (56). (E-G) ChIP-seq and DNase-seq data for mouse ES cells across the same region. Data from the ENCODE project (58).
