# Supplementary material for: Predicting the three-dimensional folding of cis-regulatory regions in mammalian genomes using bioinformatic data and polymer models
Source: Genome Biol. 2016 Mar 31;17:59. doi: 10.1186/s13059-016-0909-0 (PMC4815170; doi:10.1186/s13059-016-0909-0)
Supplement: Additional file 14 — Figure S12. A more detailed model can explain locus folding when the R2 element is deleted. (PDF 107 kb) [file 13059_2016_909_MOESM14_ESM.pdf]

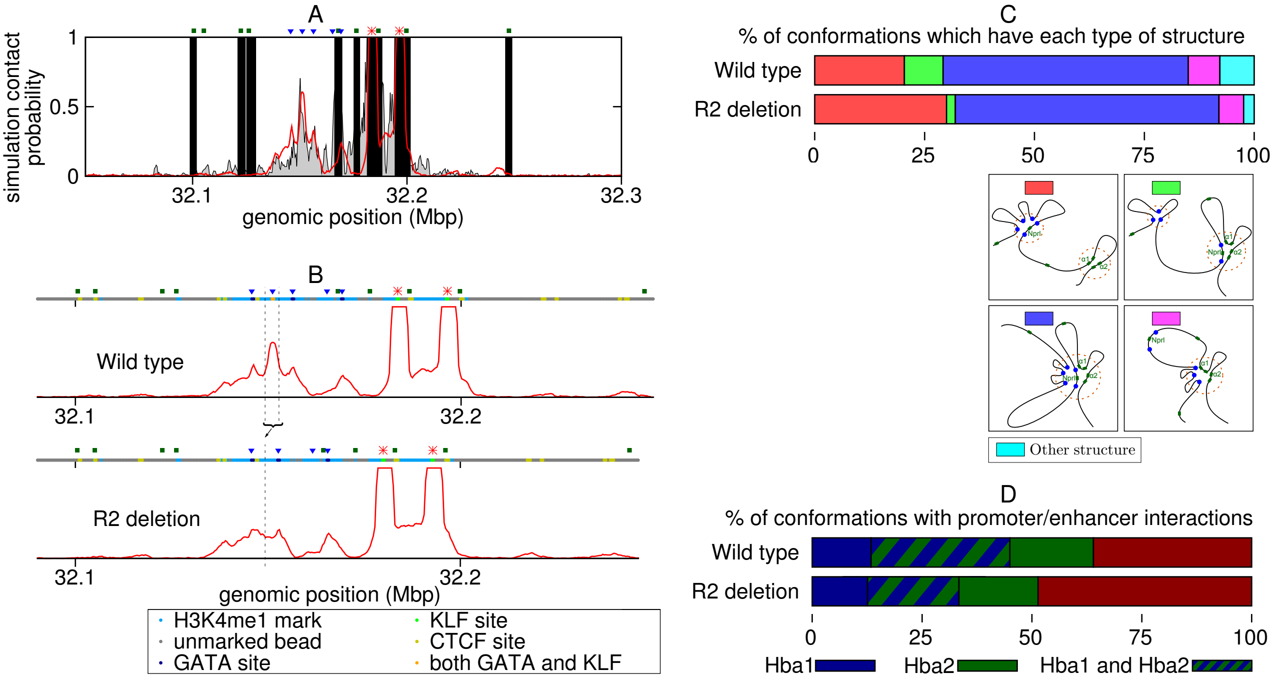

Additional file 14: Figure S12: **A more detailed model can explain locus folding when the R2 element is deleted.** (A) Plot comparing interactions with the α globin promoters in simulations with three species of bridge protein, with those from Capture-C experiments (data from Ref. [14]). Red lines show simulation results, black shaded curves the experimental data, and black bars indicate regions where no experimental data is available. The experimental data are scaled as described in Additional file 2: Supplementary Methods. The positions of the known regulatory elements and other promoters are indicated at the top of each plot with blue and green symbols respectively, and the red stars indicate the position of the Capture-C probes. The three bridge model reproduces the differences in height of interaction peaks for the regulatory elements R1-3. (B) Interactions for the α globin promoters in a wild type and a R2 knock-out simulation. Schematics indicating the binding properties of each chromatin bead are shown above the plots. The 3.6 kbp region removed in the knock-out simulation is indicated with dashed lines. (C) Plot showing the percentage of conformations which belong to each group identified by the clustering analysis, for the wild type and R2 knock-out simulations. (D) Plot showing in what percentage of conformations the two β globin gene promoters are interacting with one or more of the regulatory elements within the LCR, for the wild type and R2 knock-out simulations.
