# Supplementary material for: Predicting the three-dimensional folding of cis-regulatory regions in mammalian genomes using bioinformatic data and polymer models
Source: Genome Biol. 2016 Mar 31;17:59. doi: 10.1186/s13059-016-0909-0 (PMC4815170; doi:10.1186/s13059-016-0909-0)
Supplement: Additional file 15 — Table S1. List of all simulation parameters. (PDF 93 kb) [file 13059_2016_909_MOESM15_ESM.pdf]

Symbol	Description		Value (simulation units)
Δt	time step of numerical integration of Eq. (S1)		$0.01\ \tau_{\mathrm{LJ}}$
T	total run time of each simulation		$8 \times 10^4\ \tau_{\mathrm{LJ}}$
m_i	mass of bead i	chromatin bead	1
		protein complex	1
γ_i	friction coefficient	chromatin bead	2
		protein complex	2
d_{ii}	diameter of bead i	chromatin bead	σ
		protein complex	σ
R_0	equilibrium FENE bond length for chromatin beads		$1.6\ \sigma$
K_{FENE}	energy of the FENE bonds		$30\ k_BT$
K_{BEND}	energy of the bending interaction for the chromatin fibre		$4\ k_BT$
r_{cut}	cut-off for Lennard-Jones interaction between protein complex and protein binding chromatin bead		$1.4\ \sigma$
ε	energy for the interaction between protein complexes and binding chromatin beads		$5.39\ k_BT$ ($\varepsilon' = 10\ k_BT$)
r_{contact}	separation below which two chromatin beads are defined as being “in contact”, i.e. we assume the beads are interacting in a way analogous to that which would give a signal in a 3C experiment		$2.75\ \sigma$ (44 nm)

Additional file 15: Table S1: **List of all simulation parameters.** Parameters are given in simulation units; see Additional file 2: Supplementary Methods for details of how these relate to physical units.
