# Supplementary material for: Predicting the three-dimensional folding of cis-regulatory regions in mammalian genomes using bioinformatic data and polymer models
Source: Genome Biol. 2016 Mar 31;17:59. doi: 10.1186/s13059-016-0909-0 (PMC4815170; doi:10.1186/s13059-016-0909-0)
Supplement: Additional file 16 — Table S2. Oligonucleotide sequences for FISH probes. (PDF 68 kb) [file 13059_2016_909_MOESM16_ESM.pdf]

Probe	Oligonucleotides 5′-3′
pMPG	CTGGGGCAGACAGCCATGGTCAGTGCCCTTCCCATACTCACAGCAACCATCTGGGTGAGCgatat caagcttatcgataccgtcgac<br><br>TCAGCGAGTCGCCGGACAAGAACCTATGGGCAGTGAGTCTGCTCAGCTCAAACAGGGGCCcaccg cggtggagctccaatt
pE	AAGCATTCAGGGCTAAGGATGTAGCTTAGTAATAGAGGCCCTGAGCTCTATGACTACCACgatat caagcttatcgataccgtcgac<br><br>GAAGATGTCTCTGAATGTTCCAAGAGTTACAGTCAGTATTTCATTTAAAAATGTACATACcaccg cggtggagctccaattc
pα	CAAATTGACATGAATCAAGAATGACAACTGAGTCTTACATGGACTGTATCCAGGGTCACAgatat caagcttatcgatac<br><br>GGTATCACACACCAGGCACACATATACACATGTACGGACACATCACACACCAGGCATACATGGAC AGAAGcaccgcggtggagctccaat
p58	ATGGGCTTTATTCTCTCTGTCCCTCTGCAACACTGGTGTCACACAACACGAGTCTACCATCCTTA AAGcaccgcggtggagctccaattc<br><br>CGCCTGCAGCCAGTTCCCTTTTATACCTTTACCAACATGACTAGCTTCCTAAGCAGGGACATGga tatcaagcttatcgataccgtcgac

Additional file 16: Table S2: **Oligonucleotide sequences for FISH probes.** Sequences used to amplify the vector backbone are shown in lower case, and regions used to subclone probe sequences in upper case.
