# Supplementary material for: Predicting the three-dimensional folding of cis-regulatory regions in mammalian genomes using bioinformatic data and polymer models
Source: Genome Biol. 2016 Mar 31;17:59. doi: 10.1186/s13059-016-0909-0 (PMC4815170; doi:10.1186/s13059-016-0909-0)
Supplement: Additional file 7 — Figure S5. Simulation results show good agreement with fluorescence in situ hybridization measurements. (PDF 94 kb) [file 13059_2016_909_MOESM7_ESM.pdf]

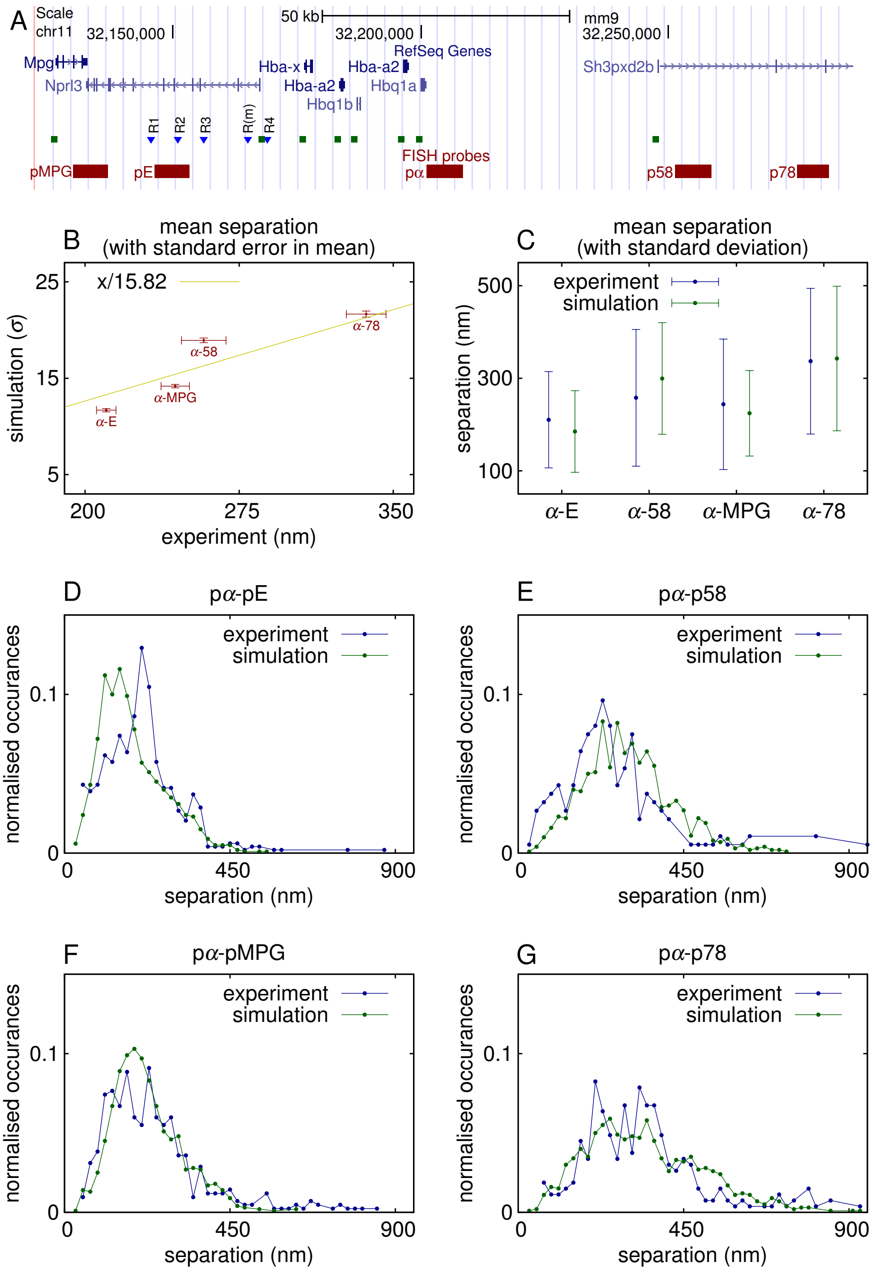

Additional file 7: Figure S5: **Simulation results show good agreement with fluorescence in-situ hybridization measurements.** (A) Genome browser view showing the locations of FISH probes across the α globin locus; the positions of the known regulatory elements are indicated with blue triangles, and the promoters are indicated with green squares. All features are shown to scale. (B) We use the mean separations of probes measured in FISH experiments to parametrise length scales in the simulations. The experiments were performed on mature erythroblasts 30 hours after differentiation, as described in Methods. Points show the experimental versus simulation mean separations with the standard error in the mean shown as error bars. The line shows a linear fit going through zero; the slope gives the conversion $\sigma = 15.95$ nm, which we round to 16 nm for the rest of the plots. (C) Plot showing the mean and standard deviation (shown as error bars) of the separation of pairs of probes. (D-G) Plots showing the full distribution of separations across many cells (at least 187 signal pairs) or simulated conformations (1000).
